# Multiple receptor tyrosine kinases regulate dengue infection of hepatocytes

**DOI:** 10.3389/fcimb.2024.1264525

**Published:** 2024-03-22

**Authors:** Natasha M. Bourgeois, Ling Wei, Nhi N. T. Ho, Maxwell L. Neal, Denali Seferos, Tinotenda Tongogara, Fred D. Mast, John D. Aitchison, Alexis Kaushansky

**Affiliations:** ^1^ Department of Global Health, University of Washington, Seattle, WA, United States; ^2^ Center for Global Infectious Disease Research, Seattle Children’s Research Institute, Seattle, WA, United States; ^3^ Department of Pediatrics, University of Washington, Seattle, WA, United States

**Keywords:** dengue (DENV), kinase signaling, neglected tropical disease, flavivirus, kinase regression, host-pathogen interactions

## Abstract

**Introduction:**

Dengue is an arboviral disease causing severe illness in over 500,000 people each year. Currently, there is no way to constrain dengue in the clinic. Host kinase regulators of dengue virus (DENV) infection have the potential to be disrupted by existing therapeutics to prevent infection and/or disease progression.

**Methods:**

To evaluate kinase regulation of DENV infection, we performed kinase regression (KiR), a machine learning approach that predicts kinase regulators of infection using existing drug-target information and a small drug screen. We infected hepatocytes with DENV *in vitro* in the presence of a panel of 38 kinase inhibitors then quantified the effect of each inhibitor on infection rate. We employed elastic net regularization on these data to obtain predictions of which of 291 kinases are regulating DENV infection.

**Results:**

Thirty-six kinases were predicted to have a functional role. Intriguingly, seven of the predicted kinases – EPH receptor A4 (EPHA4), EPH receptor B3 (EPHB3), EPH receptor B4 (EPHB4), erb-b2 receptor tyrosine kinase 2 (ERBB2), fibroblast growth factor receptor 2 (FGFR2), Insulin like growth factor 1 receptor (IGF1R), and ret proto-oncogene (RET) – belong to the receptor tyrosine kinase (RTK) family, which are already therapeutic targets in the clinic. We demonstrate that predicted RTKs are expressed at higher levels in DENV infected cells. Knockdown of EPHB4, ERBB2, FGFR2, or IGF1R reduces DENV infection in hepatocytes. Finally, we observe differential temporal induction of ERBB2 and IGF1R following DENV infection, highlighting their unique roles in regulating DENV.

**Discussion:**

Collectively, our findings underscore the significance of multiple RTKs in DENV infection and advocate further exploration of RTK-oriented interventions against dengue.

## Introduction

Dengue is a neglected tropical disease caused by dengue virus (DENV), a mosquito-borne flavivirus (Yang et al., 2021). Dengue incidence has increased at an alarming rate, with over 4.2 million dengue cases reported to the World Health Organization in 2019 compared to the 500,000 cases reported in the year 2000 (Zeng et al., 2021). Strikingly, it is estimated that hundreds of millions more dengue cases evade surveillance each year (Bhatt et al., 2013). Global warming and urbanization are expanding suitable habitats for mosquito vector populations, lending to the prediction of further escalated dengue incidence in the coming years (Anwar et al., 2019). In the absence of an effective vaccine or specific therapeutics for DENV, instances of severe disease have also been rising (Zeng et al., 2021). Identifying effective interventions against infection is imperative to combat the growing global health burden of dengue.

Efforts towards identifying compounds that directly target DENV proteins are ongoing. DENV comprises an enveloped positive-sense single-stranded RNA genome which encodes three structural proteins – envelope (Env), pre-membrane (PrM), and capsid (C) – and seven non-structural proteins – NS1, NS2A, NS2B, NS3, NS4A, NS4B, and NS5 (reviewed in (Nasar et al., 2020)). Many compounds targeting these proteins exhibit efficacy *in vitro* and *in vivo* (reviewed in (Troost and Smit, 2020)). However, none of these have demonstrated efficacy in decreasing viral load or disease in clinical trials (Tricou et al., 2010; Nguyen et al., 2013; Low et al., 2014; Whitehorn et al., 2016).

In contrast, host-targeting compounds have provided some protection against dengue disease in recent clinical trials (Palanichamy Kala et al., 2023). Importantly, targeting the host has proven to be a successful therapeutic strategy for other viruses, including human papilloma virus, hepatitis C virus, hepatitis B virus, and human immunodeficiency virus (Chaudhuri et al., 2018; Kumar et al., 2020). In addition to curbing disease in clinical trials, this strategy has also demonstrated promise for blocking DENV infection, with synergistic effects demonstrated by decreased viral load *in vitro* and *in vivo* when combining a host-targeting α-glucosidase inhibitor and the broad antiviral ribavirin, a guanosine analog (Valencia et al., 2021). However, a comprehensive understanding of druggable host molecules that are critical for successful DENV infection is unavailable, limiting the pool of candidates for host targeted intervention against dengue.

Ample evidence demonstrates that DENV relies on host factors for infection and pathogenesis, as extensively reviewed in (Roy and Bhattacharjee, 2021). For instance, DENV requires host attachment receptors and regulators of endocytosis for entry (Chen et al., 1997; Che et al., 2013; Dejarnac et al., 2018; Laureti et al., 2018; Xia et al., 2021; Liang et al., 2023), exploits regulators and structural components of host transcription and translation machinery for viral genome replication and protein production (Wang et al., 2016; Ferrari et al., 2020; Brugier et al., 2022; Krishnamoorthy et al., 2022), and manipulates factors involved in the immune response (Chen et al., 2008; Osuna-Ramos et al., 2018; Pozzi et al., 2020; Ye et al., 2021). Notably, protein kinases serve as upstream regulators of each of these events. Taken together with the existence of hundreds of kinase-targeting drugs already existing in the clinic (Roskoski, 2023), kinases are promising candidate targets for DENV therapeutics.

**Figure 1:**
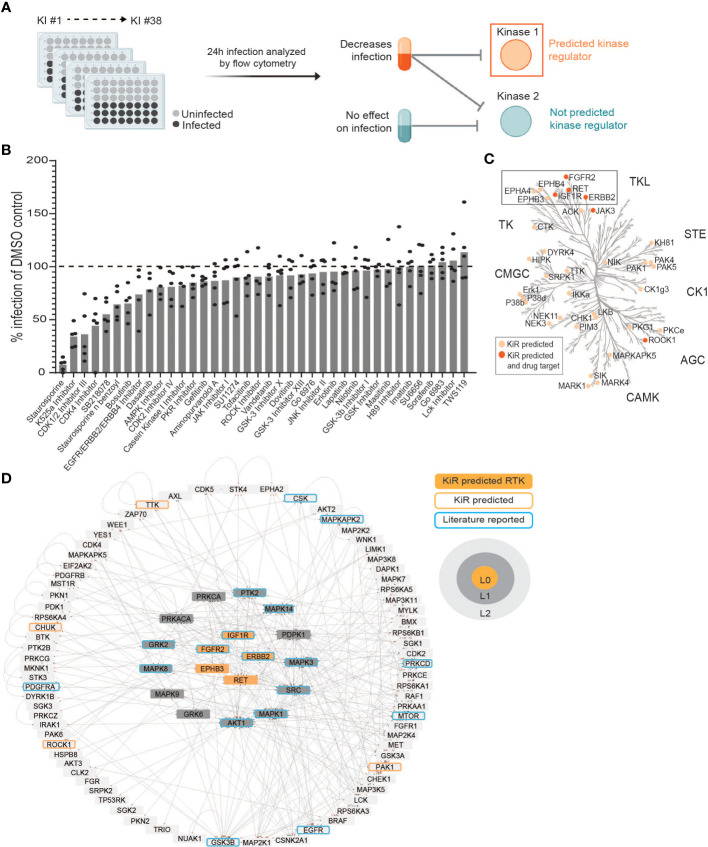
Kinase Regression (KiR) on dengue (DENV) infection predicts seven receptor tyrosine kinases (RTKs) as regulators of infection. **(A)** HepG2 cells were pre-treated with KiR kinase inhibitor (KI) panel (**Supplementary Table 1**) at a concentration of 500 nM for one hour, followed by infection with DENV2 MON601 at a MOI of 2 under continuous inhibitor exposure. 24 h post-infection, cells were fixed and stained with antibody against DENV envelope (Env) protein. Flow cytometry analysis subsequently determined the percentage of cells staining positively for Env. **(B)** Percent difference in infection rates in response to each inhibitor compared to the mock-treated control (DMSO). Data below the dashed horizontal line indicates lower infection rates than the mock control and vice versa. 5 independent biological replicates of this experiment were collected. **(C)** KiR predicted 36 kinases as potential regulators of dengue infection in HepG2s. The phylogenetic kinase family tree depicts KiR-predicted kinases and the subset that are targets of existing therapeutics in light or dark orange, respectively. **(D)** Interaction network denotes predicted RTKs (in orange), their the direct (L1, gray) or indirect (L2, light gray) interactors, other predicted kinases (orange outline), and kinases implicated in existing DENV literature (blue outline). The directionality of interactions is shown, where gray circles indicate source kinase and red arrows indicate substrate.

Evidence for the role of kinase activity in DENV infection and pathogenesis continues to accumulate (Sreekanth et al., 2018; Cortese et al., 2019; Butler et al., 2020; Carter et al., 2022; Udawatte et al., 2022), and multiple kinase inhibitors have been shown to restrict DENV infection *in vitro* and *in vivo* (Vincetti et al., 2015; Clark et al., 2016; Sreekanth et al., 2017; Pu et al., 2018; Tian et al., 2018). Despite numerous high-throughput screens aiming to identify host factors regulating DENV (Marceau et al., 2016; Savidis et al., 2016; Zhang et al., 2016; Hafirassou et al., 2017; Cortese et al., 2019; Labeau et al., 2020; Cortese et al., 2021), these attempts have failed to identify kinase regulators of infection, perhaps due to the extensive compensatory roles of other kinases or insufficient degree of depletion. Here, we overcome this shortcoming of prior screening methods by employing Kinase Regression (KiR) on DENV infection. KiR is a machine learning tool that uses a panel of 38 promiscuous kinase inhibitors – with known enzymatic inhibition activity against a total of 291 kinases spanning the human kinome – as chemical probes to screen kinases regulating infection (Anastassiadis et al., 2011; Gujral et al., 2014). KiR uses elastic net regularization to decipher which shared targets of the inhibitor panel influence infection (**Figure 1A**). KiR does not aim to assess the potential of these inhibitors as dengue treatment candidates. Rather, this approach enables investigation of predicted key regulators of infection that will ultimately inform dengue drug development. Notably, this approach has been successfully utilized to identify key host factors regulating malaria liver infection and disruption of the blood-brain barrier (Arang et al., 2017; Dankwa et al., 2021).

We performed KiR on DENV infection in HepG2 cells and predicted 36 kinase regulators. We further investigated a subset of these predictions, namely the receptor tyrosine kinases (RTKs), since many predicted kinases in this family are targeted by drugs that are already in clinical use. To validate and characterize their involvement during DENV infection, we determined the impact of DENV infection on their expression and activity, and we tested the impact of their knockdown on DENV non-structural protein 3 (NS3) and envelope protein (Env). We show that levels of KiR-predicted kinases are significantly higher in infected cells both in total and at the surface. Knockdown of a subset of these kinases significantly disrupts DENV infection, and their phosphorylation is induced at different times throughout infection. Our findings provide support for further investigation into targeting RTKs as a promising avenue for combating dengue infection and pathogenesis.

## Materials and methods

### Cell culture and maintenance

All cells used in this study were propagated from commercial passage 0 stock and stored at low passages in 10-90% FBS/10% DMSO in liquid nitrogen. Sterility of cell cultures was maintained through the use of a biosafety cabinet. Cell cultures were validated for absence of mycoplasma contamination before, during, and after experimental work using the MycoStrip™ - Mycoplasma Detection Kit (Invivogen #rep-mys). All experimental cell lines were grown under specific conditions and handled following precise procedures to ensure optimal growth and reproducibility. Typing and authentication for each cell line was provided by the American Type Culture Collection (ATCC). Each cell line was maintained for a maximum of 25 passages.

HepG2 cells were received from ATCC (#HB-8065) and grown in primocin (100 µg/mL)-supplemented Complete Hepatocyte Media (CHM): DMEM Glutamax (Gibco™ #10566016) with 10% heat-inactivated FBS (SeraPrime #F31016HI), and 4 mM L-glutamine filtered through a 0.22 µM polyethersulfone (PES) membrane. Cells were maintained in tissue culture-treated flasks at 37°C, 5% CO_2_. Upon reaching 90% confluency, cells were washed once with 1X PBS then detached with 0.25% Trypsin-EDTA (Gibco™ #25200072) for 5 min at 37°C, 5% CO_2_. Detached cells were centrifuged at 158 rcf for 3 min to remove trypsin, resuspended in primocin-supplemented CHM, then filtered through a 40 µM nylon mesh cell strainer to reduce cell clumps for improved growth and counting consistency.

Vero cells were received from ATCC (#CCL-81) and grown in primocin (100 µg/mL)-supplemented Complete Vero Media (CVM): DMEM Glutamax with 10% FBS, 1X MEM Non-Essential Amino Acid solution (NEAA, Gibco™ #11140050) and filtered through a 0.22 µM PES membrane. Cells were grown in tissue culture-treated flasks at 37°C, 5% CO_2_. Upon reaching 100% confluency, cells were washed once with 1X PBS then detached with 0.25% Trypsin-EDTA for 5 min at 37°C, 5% CO_2_. Detached cells were centrifuged at 158 rcm for 3 min to remove trypsin then resuspended in primocin-supplemented CVM.

C6/36 cells were received from ATCC (#CRL-1660) and grown in Complete C6/36 Media (CCM): MEM with 10% FBS, 1X MEM NEAA, and 100 µg/mL primocin filtered through a 0.22 µM PES membrane. Cells were grown in tissue culture-treated flasks at 28°C, 5% CO_2_. Upon reaching 100% confluency, cells were washed once with 1X PBS then detached with 0.25% Trypsin-EDTA for 5 min at 28°C, 5% CO_2_. Detached cells were centrifuged at 158 rcf for 3 min to remove trypsin then resuspended in CCM.

HEK293FT cells were a gift from Alan Aderem (Seattle Children’s Research Institute) and grown in primocin (100 µg/mL)-supplemented Complete HEK Media (CHKM): DMEM with 10% FBS, 25 mM HEPES, 1X MEM NEAA and filtered through a 0.22 µM PES membrane. Cells were grown in tissue culture-treated flasks at 37°C, 5% CO_2_. Upon reaching 100% confluency, cells were gently washed once with 1X PBS then detached with 0.025% Trypsin-EDTA (diluted in 1X PBS) for 5 min at room temperature. Detached cells were centrifuged at 158 rcf for 3 min to remove trypsin then resuspended in primocin-supplemented CHKM.

### shRNA-mediated gene knockdown

Non-replicating shRNA lentiviruses were generated in HEK293FT cells transfected with MISSION plasmids procured from Sigma-Aldrich (details in **Supplementary Table 3**). Transfection involved the combination of MISSION plasmid (6 µg), pCMV-VSV-G envelope plasmid (3 mg/ml), psPax2 packaging plasmid (6 mg/ml), and Polyethylenimine Hydrochloride (PEI Max, 1 mg/mL) in 500 µl serum free-DMEM. The resulting solution was vortexed and incubated at room temperature for 10 min before being added dropwise to HEK293FT cells at 70% confluency in 10 cm^2^ TC-treated dishes. Following overnight incubation at 37°C, 5% CO_2_, media was replaced, and cells were incubated overnight. Lentivirus-containing supernatant was harvested and filtered through a 0.45 µm PVDF membrane over the subsequent two days.

For the knock-down of host kinases of interest, HepG2 cells were reverse-transduced with the generated shRNA lentiviruses. Detached HepG2 cells were mixed with lentivirus (1 ml lentivirus-containing supernatant per 4×10^6^ cells) in antibiotic-free CHM supplemented with 1 µg/ml polybrene (EMD Millipore TR-1003-G) then plated in 10 cm^2^ TC-treated dishes. Following overnight incubation at 37°C, 5% CO_2_, cells were replenished with CHM then incubated overnight. For the next seven days, transduced cells were selected in CHM supplemented with 1 µg/ml puromycin (replenished daily). Non-transduced cells were always included in parallel as a positive control for puromycin killing. After puromycin selection, knockdown was verified at the protein level and cells were utilized for experimentation.

### Viral production, propagation, and storage

DENV2 MON601, a molecular clone of DENV-2 New Guinea strain C, was generated by transfecting *in vitro*-transcribed RNA into Vero cells (Carpp et al., 2014). Virus was propagated by interchangeably infecting 80% confluent C6/36 or Vero cell monolayers with low-passage stock virus at an MOI of 0.01 in Dengue Stock Media (DSM), comprising DMEM Glutamax supplemented with 2% FBS, 1X MEM NEAA, 25 mM HEPES, and 4 mM L-glutamine. Supernatants, collected twice weekly between 5-14 days post-infection, were cleared of cellular debris by centrifugation at 632 rcf followed by filtration through a 0.2 µM CA filter and stored at −80°C. The viral titer of stocks was enumerated as described below.

### Viral stock titer quantification

Virus stocks were titrated using a flow cytometry approach as previously described (Lambeth et al., 2005). Briefly, Vero cells were infected with serially diluted virus stocks for 24 hours, then cells were fixed. Fluorophores were conjugated to primary antibodies using Thermo Scientific™ Antibody Labeling Kits according to the manufacturers protocol. Fixed cells were stained with Env-488 and the percentage of cells that stained positive for Env-488 was obtained by flow cytometry. The titer, expressed as fluorescence forming units (FFU) per mL of virus stock, was derived from the percentage of infected cells relative to the virus volume and cell count. The titer was used to calculate the volume of virus stock necessary to achieve desired MOI.

### Viral Infection

For experimental infections, plated cells were washed once with 1X PBS then titered virus stock diluted in Opti-MEM to an MOI of 2 was added to cells at the minimum volume per well. Cells were incubated for 90 min at 37°C, 5% CO_2_ then washed once with 1X PBS. Cells were then replenished with DSM and incubated at 37°C, 5% CO_2_ for the indicated time (initial addition of virus is the start time).

### DENV detection

The pan-flavivirus envelope (Env) antibody 4G2 was prepared from hybridoma 290 supernatants and purified by protein A/G chromatography (Carter et al., 2022). Anti-Env was conjugated to Alexa Fluor 488 (Env-488) and titrated to determine the optimal concentration between 1:500 and 1:5000. Antibodies against DENV non-structural protein 3 (NS3) were obtained from GeneTex (#GTX124252), conjugated to Alexa Fluor 647 (NS3-647), and titrated as with Env-488. Fixed cells were stained with Env-488 and NS3-647 then the percentage of cells that stained positive for Env-488 or NS3-647 was obtained by flow cytometry. Fluorophores were conjugated to these primary antibodies using Thermo Scientific™ Antibody Labeling Kits according to the manufacturers protocol. Of note, some knockdown cell lines had increased background fluorescence in the channels used to measure Env-488 and NS3-647, so we included uninfected controls for each knockdown line to accurately set the infection gates.

### RTK detection

Primary antibodies against EphA4 (Thermo Scientific™ #PA5-14578), EphB3 (Santa Cruz Biotechnology, Inc. #sc-100299), EphB4 (GeneTex #GTX108595), ErbB2 (Cell Signaling Technology, Inc. (CST) # 2165), FGFR2 (CST #11835), IGF-1R (R&D Systems #MAB391) and RET (CST #3220) were used at a concentration of 1:100 then stained with anti-rabbit-PE or anti-mouse Pacific Blue secondary antibody (BioLegend^®^ #406421, Life Technologies #P10993) for flow cytometry.

For western blots, primary RTK antibodies were used at a 1:1000 concentration followed by anti-rabbit or -mouse-HRP (R&D Systems #HAF008, #HAF007) at a 1:2000 concentration. Primary antibodies p-ErbB2 (MilliporeSigma #04-293) and p-IGF-1R (MilliporeSigma #ABE332) were used at a 1:1000 concentration, p-FGFR2 (CST #3471) and p-EPHB4 (Thermo Scientific™ #PA5-64792) were used at a 1:500 concentration. Anti-mouse or anti-rabbit GAPDH were used for loading control staining at a 1:2000 concentration.

### Flow cytometry

To harvest cells for flow cytometry, HepG2s were washed once with 1X PBS then treated with TrypLE (Gibco™ #12604021) and incubated for 5 min at 37°C, 5% CO_2_. Detached cells were diluted in CHM, transferred to a 96-well U-bottom plate, then centrifuged at 158 rcf for 5 min to pellet. The supernatant was discarded, and the pellet was resuspended in 3.7% paraformaldehyde (PFA, VWR #100503-917) then incubated at room temperature on a shaker for 15 min for chemical fixation. Fixed cells were further prepared and assayed for flow cytometry as described below or stored at 4°C for no more than one week.

For analysis of total protein levels in cells, fixed cells were washed twice with 1X PBS then incubated in 0.1% Triton-X-100/1X PBS at room temperature on a shaker for 10 min. Permeabilized cells were washed twice with 1X PBS then resuspended in 0.01% Triton-X-100/2% BSA/1X PBS (GoldBio #A-420) at room temperature on a shaker for 1 h or overnight at 4°C to block non-specific protein binding. Blocked cells were pelleted at 632 rcf for 5 min and then resuspended in indicated detection antibody. Cells were stained for at least 2 h at room temperature or overnight at 4°C. For cells stained with unconjugated primary antibodies, cells were washed twice with 1X PBS after primary antibody incubation and then resuspended in secondary antibody solution for 1-2 h at room temperature. For analysis of surface protein levels in cells, fixed cells were washed twice with 1X PBS then resuspended in 2% BSA/1X PBS to block non-specific binding. After staining for kinase detection as described above, cells were permeabilized, blocked, and stained for DENV. Stained cells were washed twice with 1X PBS then assayed on an 18-color FACS analyzer harboring 405, 488, 532 and 640 nm lasers.

The collected events were analyzed using FlowJo. Events were gated for cells by size on FSC-A x SSC-A (**Supplementary Figure 1A**). Cells were gated for infection using uninfected sample stained with anti-Env-488 or anti-NS3-647 (**Supplementary Figures 1B, C**). The percentage of cells stained positive for Env-488 or NS3-647 is denoted as % infection. Kinase protein levels were obtained by adding the Geometric Mean statistic to the relevant fluorescence channel and denoted as Mean Fluorescence Intensity (MFI).

### Western blot

To collect cells for analysis by western blot, HepG2s were washed once with 1X PBS then lysed in SDS lysis buffer (50 mM Tris HCl/2% SDS/5% glycerol/5 mM EDTA/1mM NaF/dH_2_O) supplemented with cOmplete Protease Inhibitor Cocktail Tablets (Roche #11836170001), Phosphatase Inhibitor Cocktail 2 (Sigma-Aldrich^®^ #P5726), B-GP (Sigma-Aldrich^®^ #G9422), PMSF (Sigma-Aldrich^®^ #10837091001), Na_3_VO_4_ (Sigma-Aldrich^®^ #567540-5GM) and DTT (Sigma-Aldrich^®^ # D9779). Lysates were transferred to QIAshredder tubes (QIAGEN #79656) then centrifuged at 1935 rcf for 5 min to separate proteins from genetic material. Bolt Sample Reducing Agent (Invitrogen™ B0009) was added to the eluate and incubated at 70°C for 10-20 min to denature proteins.

Reduced lysates or pre-stained protein ladder (Thermo Scientific™ #26619) were loaded into the specified lanes of a Bolt 4-12% Bis-Tris Plus gel (Invitrogen™ #NW04125BOX) in Bolt™ MES SDS Running Buffer (Invitrogen™ #B0002). A 180 V current was applied for 60 min to separate proteins by size. Separated proteins were transferred onto an iBlot™ 2 Transfer Stacks PVDF membrane (Invitrogen™ IB24002) using dry transfer (as described previously (Silva and McMahon, 2014)) in an iBlot™ 2 system.

Following transfer, the PVDF membranes were incubated at room temperature on a shaker for 1 h or overnight at 4°C in 5% BSA-supplemented TBS/0.1% Tween-20 (TBST) to block non-specific protein binding during subsequent antibody staining. Blocked membranes were then incubated at 4°C overnight in primary antibody solution. Blots were then washed 3 x 5 min each in TBST and then incubated at room temperature for 1 h in secondary antibody solution.

Labeled blots were washed 4 x 5 min each then incubated in SuperSignal™ West Pico PLUS Chemiluminescent Substrate (Thermo Scientific™ #34577) at room temperature for 3 min. Developed blots were scanned for chemiluminescence on a BioRad ChemiDoc Imaging System. ImageJ was used to enhance raw images for display and to quantify band intensity. Band intensity is reported as chemiluminescence of the protein of interest, normalized as indicated.

### Prediction of kinases

HepG2 cells were pre-treated with the KiR inhibitor panel in Opti-MEM (**Supplementary Table 1**) at 500 nM for 1 h, after which media was removed, and cells were replenished with either DSM or DENV2 MON601 at an MOI of 2 in the presence of the inhibitor panel. After 90 min, cells were washed once with 1X PBS then replenished with inhibitor-supplemented DSM. 24 h post-infection, cells were fixed and stained with Env-488. The percentage of cells staining positive for Env was quantified by flow cytometry. Inhibitor-induced background fluorescence was measured on uninfected, inhibitor-treated samples and subtracted from infection values. Resulting values below zero were adjusted to zero. Grubb’s Test was used to remove outlier data.

The elastic net regularization algorithm used for this study was published previously (Dankwa et al., 2021; Wei et al., 2022). Briefly, normalized percent infection data from five independent kinase inhibitor screens and existing biochemical data of the kinase inhibitors against 300 recombinant protein kinases (Anastassiadis et al., 2011), were input into the elastic net regularization algorithm using a condition-specific cross-validation strategy. The glmnet package (version 2.2.1, https://github.com/bbalasub1/glmnet_python) in Python (version 3.7.6, https://www.python.org) was used for performing elastic net regression, with the elastic net mixing parameter α, which confers the stringency of model selection, scanned from 0.1 to 1.0 in steps of 0.1. Predictions using an α of 0.8 are reported in this manuscript. The regularization path was computed for the elastic net penalty at a grid of values for the regularization parameter λ of 10^3^. The Python code used for this analysis is provided in Supplementary Files (**Supplementary File 1**).

### Building kinase interaction networks

Phosphosignaling networks were built to identify upstream/downstream kinases of the KiR predicted kinases and infer the connections between them. Searches for two layers upstream/downstream of the KiR predicted kinases were done using the kinase-substrate phosphorylation database PhosphoSitePlus^®^ (Hornbeck et al., 2015). These data were visualized using Cytoscape 3.9.1 (Shannon et al., 2003). L0, L1, and L2 nodes were manually spatially organized, then the degree-sorted circular layout algorithm was applied to spatially organize predicted RTKs and their interactions based on degree of connection.

### Statistical analyses

Sample set details and method of statistical analysis are reported for each experiment in the corresponding figure legend. For instances where statistical analyses are not included, raw data is available in the **Supplementary Information**.

## Results

### Kinase regression predicts multiple receptor tyrosine kinases that regulate DENV infection of hepatocytes

The liver is directly infected by DENV and can be critically damaged in severe dengue cases (reviewed in (Chia et al., 2020)). Additionally, kinase regulation of dengue-induced liver injury has been reported (Sreekanth et al., 2017; Sreekanth et al., 2020). Considering this, we chose to apply kinase regression (KiR) to DENV infection of HepG2 hepatoma cells, a widely used model for studying DENV infection of the liver. We pre-treated HepG2 cells with a panel of 38 inhibitors collectively targeting 291 kinases (**Supplementary Table 1**) with known target overlap at 500 nM. To robustly analyze inhibition data for dengue infection using KiR, we chose to match the concentration used to produce the biochemical inhibition data that informs KiR (Anastassiadis et al., 2011); cytotoxic effects were not quantified. An hour after treatment, we infected the cells with DENV2 MON601 at an MOI of 2 and continued inhibitor treatment. Twenty-four hours post-infection (hpi), we fixed the cells and stained them with a 488-conjugated Env antibody solution. The percentage of cells staining positive for Env was quantified by flow cytometry (**Figure 1A**). Four inhibitors decreased DENV infection by over 50%, while several inhibitors led to more modest decreases in DENV infection. In contrast, three inhibitors had no measurable effect on DENV infection, and three inhibitors led to modest increases in DENV infection (**Figure 1B**). Rather than interpreting the effect of any individual inhibitor, we systematically predicted kinases important for DENV infection by inputting these data, along with existing information on kinase-substrate inhibition, into an elastic net regularization algorithm (see Methods) (Anastassiadis et al., 2011; Gujral et al., 2014). This analysis led us to identify 36 kinases potentially crucial in regulating dengue infection (**Figure 1C**, **Supplementary Table 2**).

To prioritize predictions for subsequent investigation, we explored which of the predicted kinases are targets of FDA approved drugs. Strikingly, the majority of predicted kinases with available drugs were receptor tyrosine kinases (RTKs), despite the fact that RTKs represent only about 10% of all kinases (**Figure 1C**) (Salokas et al., 2022). The predicted RTKs included Ephrin type-A receptor 4 (EPHA4), Ephrin type-B receptor 3 (EPHB3), Ephrin type-B receptor 4 (EPHB4), Receptor tyrosine-protein kinase erbB-2 (ERBB2), Fibroblast growth factor receptor 2 (FGFR2), Insulin-like growth factor 1 receptor (IGF1R), and Proto-oncogene tyrosine-protein kinase receptor Ret (RET).

To further assess the potential of RTKs as regulators of DENV infection, we examined if the seven predicted RTKs interacted with other KiR predictions or with kinases previously shown to be important for DENV infection (Chu and Yang, 2007; Ceballos-Olvera et al., 2010; Shyu et al., 2010; Chang et al., 2012; Le Sommer et al., 2012; Chen et al., 2013; de Wispelaere et al., 2013; Li et al., 2013; Nagila et al., 2013; Yeh et al., 2013; Liu et al., 2014; Sreekanth et al., 2014; Callaway et al., 2015; Kumar et al., 2016; Sreekanth et al., 2016; Noppakunmongkolchai et al., 2016; Duran et al., 2017; Jordan and Randall, 2017; Limjindaporn et al., 2017; Smith et al., 2017; Airo et al., 2018; Barbachano-Guerrero et al., 2020; Cuartas-López and Gallego-Gómez, 2020; de Oliveira et al., 2020; Kong et al., 2020; Lahon et al., 2021; Liu et al., 2021; Carter et al., 2022; Puerta-Guardo et al., 2022; Rahman et al., 2022; Sinha et al., 2022; Wu et al., 2022; Kao et al., 2023). We utilized the kinase-substrate phosphorylation database PhosphoSitePlus^®^ (Hornbeck et al., 2015) to identify interacting kinases and substrates of the predicted RTKs and then visualized these interactions using Cytoscape 3.9.1 (**Figure 1D**). Known interactions for EPHA4 and EPHB4 are entirely composed of autophosphorylation events within the PhosphoSitePlus^®^ database, so they are not included in this network. We observed that EPHB3, ERBB2, FGFR2, IGF1R, and RET are upstream regulators both of kinases shown to be important in previous dengue research and of kinases predicted by KiR. We therefore hypothesized that the KiR-predicted RTKs are key mediators of DENV infection.

### Elevated levels of RTKs are observed in DENV-infected cells

RTKs are stationed on the plasma membrane of cells, where they mediate growth factor signaling and cell-cell communication (reviewed in (Trenker and Jura, 2020)). After interacting with ligand, RTKs are often endocytosed and activate signaling cascades that orchestrate cell function. There is extensive evidence that viruses can alter canonical RTK expression to hijack endocytosis or manipulate replication and cell death machinery, as reviewed in (Lateef and Wise, 2018). We thus investigated whether the KiR predicted RTKs – EPHA4, EPHB3, EPHB4, ERBB2, FGFR2, IGF1R, and RET – were present at higher levels in infected cells.

**Figure 2:**
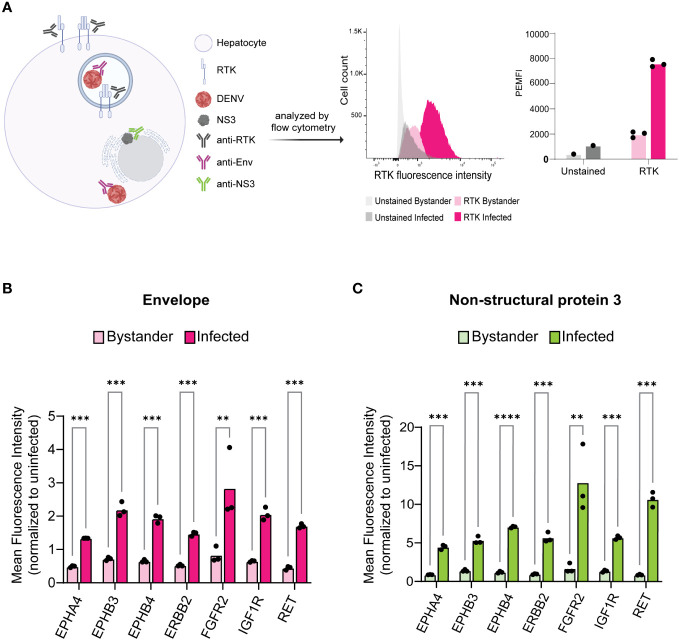
Infected cells exhibit increased levels of KiR-predicted RTKs. HepG2 cells were infected with DENV at an MOI of 2 for 24 h then analyzed for protein levels of predicted RTKs by flow cytometry. **(A)** Diagram of detection method for RTK expression. HepG2 cells were permeabilized to simultaneously probe for total protein levels of RTK and DENV Env or NS3. Plots on the right show a representative histogram and quantification for RTK expression analysis employed: fluorescence intensity of PE-tagged RET is shown in Env- (bystander) versus Env+ (infected) cells. Cells unstained for RTK are included as a staining control. **(B)** Difference in mean fluorescence intensity (MFI) of each RTK between Env- and Env+ cells or **(C)** NS3- (bystander) and NS3+ (infected) cells. Significance of the differences are indicated by Student’s t-test as denoted by asterisks, where ** = p-value<0.005 and *** = p-value<0.0005.

We infected HepG2 cells with DENV2 MON601 for 24 h and then stained them with Phycoerythrin (PE)- or Pacific Blue-labeled RTK antibodies in parallel with Env-AlexaFluor 488 and NS3- AlexaFluor 647; RTK-unstained samples were included as a control (**Figure 2A**). The mean fluorescence intensity (MFI) of each labeled RTK was first analyzed in uninfected cells to establish the baseline expression of each RTK. We then quantified the MFI of the Env- (bystander) and the Env+ (infected) cell populations and normalized these data to the MFI of uninfected cells for each RTK (**Figure 2B**). The same analysis was performed on NS3+ infected cells and NS3- bystander cells (**Figure 2C**). Interestingly, we found that the level of each RTK was significantly higher in infected cells compared to bystander cells. These results were consistent across biological replicates with varied infection rates (**Supplementary Figure 2**).

### Surface levels of RTKs are also elevated in HepG2 cells infected with DENV

**Figure 3:**
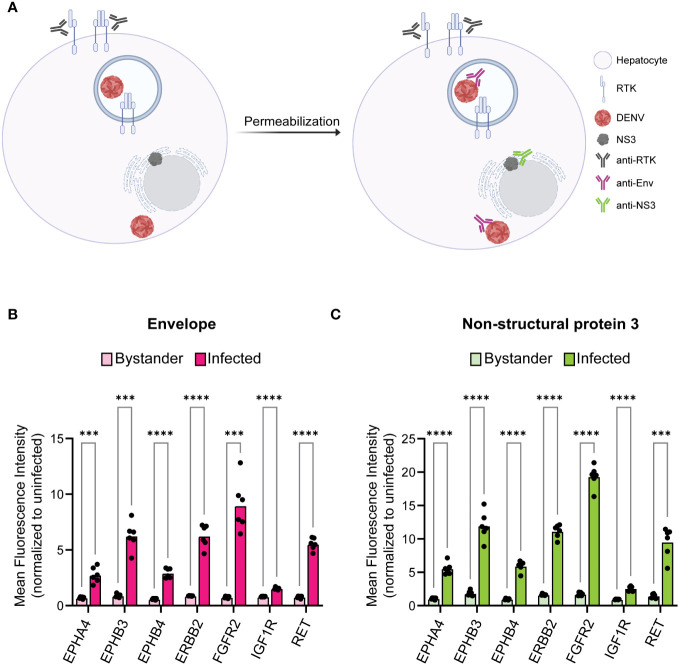
Infected cells have increased surface expression of predicted RTKs. HepG2 cells were infected with DENV at a MOI of 2 for 24 h then analyzed for protein levels of predicted RTKs by flow cytometry. **(A)** Diagram of detection method for RTK expression. HepG2 cells were probed for surface protein levels of RTK then permeabilized to probe for DENV Env or NS3. **(B)** Difference in MFI of each RTK between Env- (bystander) and Env+ (infected) cells or **(C)** NS3- (bystander) and NS3+ (infected) cells. Significance of the differences are indicated by Student’s t-test as denoted by asterisks, where *** = p-value<0.0005 and **** = p-value<0.00005.

RTKs can regulate infection either on the surface as a mediator of viral endocytosis or within the cell as a signal transducer. This led us to investigated whether RTK expression specifically at the cell surface was altered in infected cells. We probed 24 hr DENV-infected HepG2 cells for RTK prior to permeabilization such that only surface exposed RTK would be measured (**Figure 3A**). We compared the RTK MFI of bystander and infected cells for DENV Env (**Figure 3B**, **Supplementary Figure 3A**) and NS3 (**Figure 3C**, **Supplementary Figure 3B**). We found that, similar to what was observed for total RTK levels, the amount of RTK specifically at the surface was significantly increased in infected cells compared to bystander cells.

### Knockdown of EPHB4, ERBB2, FGFR2, or IGF1R impairs DENV infection

**Figure 4:**
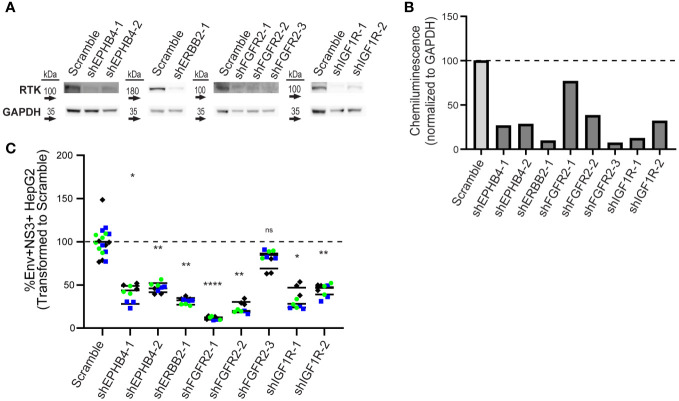
Knockdown of EPHB4, ERBB2, FGFR2, and IGF1R results in decreased DENV infection. HepG2 cells were transduced with shRNA lentivirus targeting KiR-predicted RTKs then analyzed for protein reduction by western blot **(A, B)**. Viable cells with successful knockdown in protein expression were infected with DENV2 MON601, and the percent change of Env and NS3 double positive cells is shown **(C)**. Data represent three independent infections on a single transduction per clone, where green circle, blue square, and black diamond indicate each independent infection. Horizontal black bar represents the average of all replicates for each condition. Significance of differences in infection between knockdown lines and scramble were analyzed by Student’s t-test and are indicated on the graph, where ns = non-significant, * = p-value<0.05, ** = p-value<0.05, *** = p-value<0.0005, and **** = p-value<0.00005.

Higher levels of RTK does not necessarily translate to a functional role, so we next determined if genetically reducing RTK levels affected DENV infection. We generated lentivirus clones carrying a puromycin selection marker and shRNA against each RTK, or scrambled shRNA, as a control (**Supplementary Table 3**). We transduced HepG2 cells with each lentivirus clone individually and selected for knockdown cells with puromycin for seven days to generate RTK knockdown lines. Knockdown lines with <10% loss in viability were analyzed by western blot to assess protein knockdown. We successfully obtained one or more knockdown lines for EPHB4, ERBB2, FGFR2, and IGF1R (**Figures 4A, B**), but we were unable to procure knockdown lines for EPHA4, EPHB3, or RET, due to either loss in viability or observing no reduction in protein levels after lentiviral transduction.

To test whether reduced protein levels of EPHB4, ERBB2, FGFR2, or IGF1R impacted infection, we infected control and knockdown lines with DENV2 MON601 for 24 h. We then quantified the percentage of Env and NS3 double positive cells by flow cytometry. We calculated infection in each knockdown line as a percentage of the scramble control. Remarkably, all knockdown lines had significantly fewer infected cells, except for one FGFR2 line (**Figure 4C**, **Supplementary Figure 4**). Thus, EPHB4, ERBB2, IGF1R, and FGFR2 are essential for optimal dengue infection, although interpreting the role of FGFR2 is challenging due to the FGFR2-3 clone having a different phenotype than the other two clones.

### DENV infection induces changes in ERBB2 and IGF1R phosphorylation

We next investigated if DENV infection impacts phosphorylation of the RTKs whose knockdown reduced infection. In particular, we were interested in RTK activation at different phases of the viral cycle, since different types of RTK therapeutics could be utilized depending on the role of the RTK. For instance, monoclonal antibodies could be employed to block a binding interaction at the surface during viral entry, while small molecule inhibitors could block pathway activation essential for later in the viral life cycle. To this end, we infected HepG2 cells with DENV2 MON601 and collected cell lysates at various time increments throughout infection: 0.5 – 1.5 hpi to investigate activity during viral binding and entry, 6 – 8 hpi for during viral replication, and 16 – 24 hpi to monitor re-infection (Suksanpaisan et al., 2009). We probed cell lysates for phospho (p-) ERBB2 (Thr686), p-IGF1R (Tyr1161/Tyr1165/Tyr1166), p-FGFR2 (Tyr653/654), and p-EPHB4 (Tyr987); however, we did not detect a robust and quantifiable signal for p-FGFR2 or p-EPHB4 in infected or uninfected cells (data not shown). Lysates were also probed for total (t-) ERBB2 and t-FGFR2, then the phosphorylation of ERBB2 and FGFR2 were quantified as a ratio of phosphorylated protein over total protein. Infection was also monitored at each time point to ensure robust infection (**Supplementary Figure 5**).

**Figure 5:**
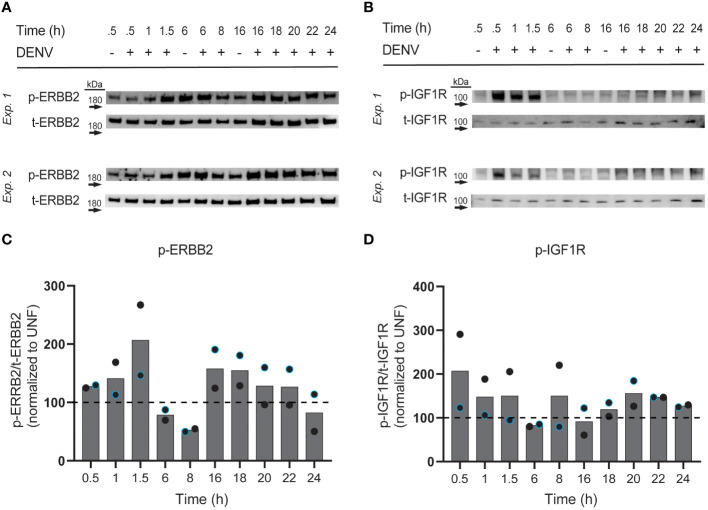
Phosphorylation of ERBB2 and IGF1R is induced by DENV infection. HepG2 cells were infected with DENV2 MON601 then lysed at the indicated time-points. Lysates were analyzed for phospho (p) and total (t) -ERBB2 **(A)** and p- and t-IGF1R **(B)** levels by western blot. The ratio of p-ERBB2 to t-ERBB2 and p-IGF1R to t-IGF1R was calculated. Percent change in the ratio of phospho to total RTK between infected samples and appropriate uninfected control sample is shown [0.5 – 1.5 hpi were compared to uninfected 0.5 h sample, 6 – 8 hpi to uninfected 6 h sample, and 16-24 hpi to uninfected 16 h sample, based on preliminary experimentation (data not shown)] **(C, D)**. Data represent two independent experiments, where the black circle is one experiment and the blue-outlined circle is another. Dashed line demarks the uninfected control.

We observed altered levels of phosphorylated ERBB2 and IGF1R with differing kinetic patterns during infection (**Figures 5A–C**). Interestingly, phosphorylated ERBB2 (p-ERBB2) increased gradually from 0.5 to 1.5 hpi on average, having a greater than 2-fold increase at 1.5 hpi, but was then suppressed at 6 and 8 hpi. p-ERBB2 was increased at 16 hpi but then gradually declined through 24 hpi. In contrast, phosphorylated IGF1R was induced 2-fold at 0.5 hpi then modestly elevated at other time-points, except for at 6 and 16 hpi, when p-IGF1R was suppressed. These kinetic differences in RTK activation suggest that different RTKs have roles at distinct stages of DENV infection in hepatocytes. Together, we see that ERBB2 and IGF1R are subject to changes in phosphorylation throughout the DENV life cycle.

## Discussion

Dengue poses a significant global health threat. To mitigate this, we sought to identify host regulators of DENV infection to inform therapeutic interventions for DENV infection. We chose to focus on kinases in our study, as there is an extensive library of pharmacological tools to modulate their activity in the clinic and thus could provide useful therapeutic targets for dengue. Using kinase regression (KiR), an approach consisting of a small kinase inhibitor screen and linear regression modeling, we predicted 36 kinases that could regulate DENV infection in HepG2 cells (**Figures 1A–C**). These 36 kinases, likely significant in various cell types due to the universality of kinase networks, represent potential targets for dengue interventions.

We narrowed our focus to a subset of the KiR-predicted kinases, the receptor tyrosine kinases (RTKs) – EPHA4, EPHB3, EPHB4, ERBB2, FGFR2, IGF1R, and RET. Many of the KiR-predicted RTKs are targets of drugs already used in the clinic (**Figure 1C**), and they have been shown to interact with other regulators of dengue infection (**Figure 1D**). Notably, the PhosphoSitePlus^®^ database used to generate **Figure 1D** does not exhaustively catalog all kinase-substrate interactions, so additional interactions beyond those illustrated in **Figure 1D** likely exist. For instance, EPHA4 is known to stimulate signaling through RAC-alpha serine threonine-protein kinase (AKT), a regulator of dengue infection, and EPHB4 stimulates KiR-predicted rho associated coiled-coil containing protein kinase 1 (ROCK1) (Baudet et al., 2020; Frye et al., 2020). Building a more comprehensive network utilizing multiple protein interaction databases could provide systematic insight into the involvement of predicted kinases.

Interestingly, we discovered that DENV-infected HepG2 cells have elevated expression of both total and surface RTK (**Figures 2**, **3**). A limitation of this approach is that there is background fluorescence in unstained cells that may convolute the quantification of receptor levels. To address this, we analyze the fluorescence intensity relative to an uninfected sample for each RTK, rather than making conclusions about the absolute value of fluorescence intensity. Given this limitation, and the fact that abundance measures alone cannot verify a functional role in infection, we investigated the impact of RTK knockdown on DENV infection. We observed that depletion of EPHB4, ERBB2, FGFR2, or IGF1R protein led to reduced levels of Env and NS3 double positive cells, suggesting that these RTKs can be targeted to interrupt DENV infection (**Figure 4**, **Supplementary Figure 4**). However, there are limitations to these data, particularly the differing phenotype observed across FGFR2 knockdown lines. While FGFR2-1 and -3 knockdown lines demonstrated reduced infection, FGFR2-3 did not. Thus, it is challenging to understand the importance of FGFR2 during dengue infection. It is possible that the FGFR2-3 shRNA clone results in a different FGFR2 mRNA isoform which produces a truncated protein product that can still accomplish interaction with DENV but is not captured by the antibody we used to determine protein knockdown. Notably, aligning the FGFR2 shRNA clone sequences (**Supplementary Table 3**) with FGFR2 reference sequence shows that each of these clones targets a different exon of the FGFR2 gene, with FGFR2-1 targeting Exon 19, FGFR2-2 targeting Exon 3, and FGFR2-3 targeting Exon 10. Further investigation into the impact of each shRNA clone on FGFR2 protein structure and function could help clarify its role during infection. The absence of additional ERBB2 knockdown lines or knockdown lines with pooled shRNA clones also limits the conclusions that can be made about the necessity of these RTKs in DENV infection. More robust conclusions could be made if each RTK is assessed by multiple clones, and if pooling clones cumulatively affects infection. Furthermore, additional work investigating the impact of RTK knockdown on entry as well as on the production of infectious progeny could provide more support for their role during infection and give insight into their role during DENV infection. Strikingly, we observe DENV infection leads to phosphorylation of ERBB2 and IGF1R at different times during infection (**Figure 5**). However, interpretation of these data is limited since three representative uninfected control samples were chosen rather than including an uninfected sample at each time point. Nevertheless, one hypothesis that can be drawn from these data is that IGF1R activity is solely important early during infection, while ERBB2 has roles throughout the infection. p-ERBB2 is highest at 1.5 hpi, while p-IGF1R is highest at 0.5 hpi; this could be due to a role for ERBB2 later during endocytosis while IGF1R is involved earlier, during binding. Interestingly, p-ERBB2 and p-IGF1R are suppressed at 6 hpi. It is possible that viral infection disrupts regulators of RTK recycling or directly interacts with RTK to disrupt activation. Investigating the temporal phosphorylation activity of the substrates of these RTKs could provide more functional insight into their role on DENV infection.

Ultimately, these data support further investigation into KiR-predicted RTKs as targets for dengue therapeutics. RTK-targeting drugs are already used as first- or second-line drugs for cancer, in some cases with limited toxicity. Additionally, combining RTK therapeutics with direct-acting antivirals could enable even lower, less cytotoxic doses to be used. Inhibiting RTK receptor activity at the point of entry or the subsequent RTK-driven signaling cascades could block DENV infection. Further investigation will inform which RTK-targeting interventions could be useful in disrupting dengue infection.

To this end, differential temporal kinetics of ERBB2 and IGF1R activation during DENV infection is particularly intriguing given that our data indicated that no single kinase knockdown or kinase inhibitor can, on its own, completely abolish DENV infection of HepG2 cells (**Figures 1**, **4**), and previous studies have demonstrated that combination kinase inhibitor treatment leads to increased efficacy against DENV (Bekerman et al., 2017; Pu et al., 2018). Therefore, combination treatment to block both IGF1R and ERBB2 could result in more robust inhibition than targeting either one individually. Of note, IGF1R and ERBB2 drug combinations are already being investigated in the context of cancer (Chakraborty et al., 2015; McDermott et al., 2017). Additionally, both IGF1R and ERBB2 interact with other host factors known to play a role in dengue infection or pathogenesis, such as phosphoinositide 3-kinase (PI3K)/AKT (Liu et al., 2014; Chen et al., 2017; Okamoto et al., 2017; Airo et al., 2018; Cuartas-López et al., 2018; Lahon et al., 2021; Liu et al., 2021; Carter et al., 2022), proto-oncogene tyrosine-protein kinase Src (Chu and Yang, 2007; Chu and Yang, 2008; Vincetti et al., 2015; Kumar et al., 2016), and mitogen-activated protein kinases (ERK) (Shyu et al., 2010; He et al., 2012; de Oliveira et al., 2020; Wu et al., 2022; Chen et al., 2023; Liang et al., 2023). Thus, the activity of ERBB2 and IGF1R during DENV infection should be further investigated to understand their utility as dual targets of dengue therapeutics.

In addition to small molecule inhibitors, RTKs can also be targeted by monoclonal antibodies in the clinic (Pelletier and Mukhtar, 2020; Hwang et al., 2021). Elevated surface levels of RTKs in infected cells (**Figure 3**) suggests that they could have roles as receptors for viral entry, in which case blocking receptor interaction would inhibit DENV infection. If further investigation demonstrates their role as entry receptors, approved monoclonal antibodies such as Teprotumumab (IGF1R) and Trastuzumab (ERBB2) could prove useful for blocking DENV infection.

Importantly, since kinase inhibitors have the propensity to inhibit multiple kinases through polypharmacology, single kinase inhibitors that block multiple kinase regulators of dengue should also be explored. For instance, FGFR inhibitor Futibatinib has the potential to block both FGFR2 activity demonstrated in the present study (**Figure 4**) in addition to FGFR4 activity previously shown to regulate DENV infection (Cortese et al., 2019). Promiscuous inhibitors targeting a combination of kinases identified in this study and others could enhance anti-DENV activity without generating toxicity often observed in multi-drug regimens.

While we focused on only a subset of predicted kinases, the RTKs, for experimental follow-up and discussion as therapeutic candidates, the remaining predictions are also promising as druggable regulators of DENV infection. One important mechanism that should be explored is how downstream targets of KiR predicted kinases (**Figure 1D**) may mediate detrimental DENV immune responses which could be therapeutically blocked with RTK-targeting drugs. For instance, KiR predicted liver kinase B1 (LKB1) is part of the regulation pathway for alanine transaminase (ALT), a liver enzyme that is significantly elevated in severe dengue cases (Yuan et al., 2022). Inhibitor of nuclear factor kappa-B kinase subunit alpha (IKK-α) and NF-kappa-B-inducing kinase (NIK) are upstream regulators of TNF which is implicated in DENV pathogenesis (Bhatt et al., 2021). Additionally, ROCK1, which is a target of Fasudil – used in the clinic for cerebral vasospasm – is known to regulate the migration and adhesion of inflammatory cells (Singh et al., 2018). These examples highlight pathways that could be targeted to block infection while simultaneously preventing immune-mediated disease. The network analysis we utilize provides a framework for forming such hypotheses on the systematic mechanism of kinase involvement.

In conclusion, this study provides novel insight into kinase regulators of DENV infection and highlights the potential of receptor tyrosine kinases as therapeutic targets against dengue.

## Data availability statement

The original contributions presented in the study are included in the article/**Supplementary Material**. Further inquiries can be directed to the corresponding author.

## Author contributions

NB: Conceptualization, Methodology, Data curation, Formal analysis, Investigation, Software, Validation, Visualization, Writing – original draft. LW: Data curation, Formal analysis, Methodology, Resources, Writing – review & editing. NH: Data curation, Writing – review & editing. MN: Writing – review & editing, Conceptualization, Formal analysis. DS: Conceptualization, Writing – review & editing. TT: Conceptualization, Writing – review & editing, Data curation. FM: Conceptualization, Writing – review & editing. JA: Conceptualization, Writing – review & editing, Methodology, Supervision. AK: Conceptualization, Methodology, Supervision, Writing – review & editing, Funding acquisition, Project administration, Resources.

## References

[B1] Airo A. M. Urbanowski M. D. Lopez-Orozco J. You J. H. Skene-Arnold T. D. Holmes C.. (2018). Expression of flavivirus capsids enhance the cellular environment for viral replication by activating Akt-signalling pathways. Virology 516, 147–157. doi: 10.1016/j.virol.2018.01.009 29358114

[B2] Anastassiadis T. Deacon S. W. Devarajan K. Ma H. Peterson J. R. (2011). Comprehensive assay of kinase catalytic activity reveals features of kinase inhibitor selectivity. Nat. Biotechnol. 29, 1039–1045. doi: 10.1038/nbt.2017 22037377 PMC3230241

[B3] Anwar A. Khan N. Ayub M. Nawaz F. Shah A. Flahault A. (2019). Modeling and predicting dengue incidence in highly vulnerable countries using panel data approach. Int. J. Environ. Res. Public Health 16 (13), 2296. doi: 10.3390/ijerph16132296 31261672 PMC6650977

[B4] Arang N. Kain H. S. Glennon E. K. Bello T. Dudgeon D. R. Walter E. N. F.. (2017). Identifying host regulators and inhibitors of liver stage malaria infection using kinase activity profiles. Nat. Commun. 8, 1232. doi: 10.1038/s41467-017-01345-2 29089541 PMC5663700

[B5] Barbachano-Guerrero A. Endy T. P. King C. A. (2020). Dengue virus non-structural protein 1 activates the p38 MAPK pathway to decrease barrier integrity in primary human endothelial cells. J. Gen. Virol. 101, 484–496. doi: 10.1099/jgv.0.001401 32141809

[B6] Baudet S. Bécret J. Nicol X. (2020). Approaches to manipulate ephrin-A:EphA forward signaling pathway. Pharmaceuticals 13, 140. doi: 10.3390/ph13070140 32629797 PMC7407804

[B7] Bekerman E. Neveu G. Shulla A. Brannan J. Pu S. Y. Wang S.. (2017). Anticancer kinase inhibitors impair intracellular viral trafficking and exert broad-spectrum antiviral effects. J. Clin. Invest. 127, 1338–1352. doi: 10.1172/JCI89857 28240606 PMC5373883

[B8] Bhatt P. Sabeena S. P. Varma M. Arunkumar G. (2021). Current understanding of the pathogenesis of dengue virus infection. Curr. Microbiol. 78, 17–32. doi: 10.1007/s00284-020-02284-w 33231723 PMC7815537

[B9] Bhatt S. Gething P. W. Brady O. J. Messina J. P. Farlow A. W. Moyes C. L.. (2013). The global distribution and burden of dengue. Nature 496, 504–507. doi: 10.1038/nature12060 23563266 PMC3651993

[B10] Brugier A. Hafirrassou M. L. Pourcelot M. Baldaccini M. Kril V. Couture L.. (2022). RACK1 associates with RNA-binding proteins vigilin and SERBP1 to facilitate dengue virus replication. J. Virol. 96, e0196221. doi: 10.1128/jvi.01962-21 35266803 PMC9006918

[B11] Butler M. Chotiwan N. Brewster C. D. DiLisio J. E. Ackart D. F. Podell B. K.. (2020). Cyclin-dependent kinases 8 and 19 regulate host cell metabolism during dengue virus serotype 2 infection. Viruses 12 (6), 654. doi: 10.3390/v12060654 32560467 PMC7354599

[B12] Callaway J. B. Smith S. A. McKinnon K. P. de Silva A. M. Crowe J. E. Jr. Ting J. P. (2015). Spleen tyrosine kinase (Syk) mediates IL-1β Induction by primary human monocytes during antibody-enhanced dengue virus infection. J. Biol. Chem. 290, 17306–17320. doi: 10.1074/jbc.M115.664136 26032420 PMC4498069

[B13] Carpp L. N. Rogers R. S. Moritz R. L. Aitchison J. D. (2014). Quantitative proteomic analysis of host-virus interactions reveals a role for Golgi brefeldin A resistance factor 1 (GBF1) in dengue infection. Mol. Cell Proteomics 13, 2836–2854. doi: 10.1074/mcp.M114.038984 24855065 PMC4223476

[B14] Carter C. C. Mast F. D. Olivier J. P. Bourgeois N. M. Kaushansky A. Aitchison J. D. (2022). Dengue activates mTORC2 signaling to counteract apoptosis and maximize viral replication. Front. Cell. Infection Microbiol. 12. doi: 10.3389/fcimb.2022.979996 PMC9510660 36171757

[B15] Ceballos-Olvera I. Chávez-Salinas S. Medina F. Ludert J. E. del Angel R. M. (2010). JNK phosphorylation, induced during dengue virus infection, is important for viral infection and requires the presence of cholesterol. Virology 396, 30–36. doi: 10.1016/j.virol.2009.10.019 19897220

[B16] Chakraborty A. K. Zerillo C. DiGiovanna M. P. (2015). *In vitro* and in *vivo* studies of the combination of IGF1R inhibitor figitumumab (CP-751,871) with HER2 inhibitors trastuzumab and neratinib. Breast Cancer Res. Treat 152, 533–544. doi: 10.1007/s10549-015-3504-2 26195122

[B17] Chang T. H. Chen S. R. Yu C. Y. Lin Y. S. Chen Y. S. Kubota T.. (2012). Dengue virus serotype 2 blocks extracellular signal-regulated kinase and nuclear factor-κB activation to downregulate cytokine production. PLoS One 7, e41635. doi: 10.1371/journal.pone.0041635 22927911 PMC3425550

[B18] Chaudhuri S. Symons J. A. Deval J. (2018). Innovation and trends in the development and approval of antiviral medicines: 1987-2017 and beyond. Antiviral Res. 155, 76–88. doi: 10.1016/j.antiviral.2018.05.005 29758235 PMC7126013

[B19] Che P. Tang H. Li Q. (2013). The interaction between claudin-1 and dengue viral prM/M protein for its entry. Virology 446, 303–313. doi: 10.1016/j.virol.2013.08.009 24074594

[B20] Chen H. H. Chen C. C. Lin Y. S. Chang P. C. Lu Z. Y. Lin C. F.. (2017). AR-12 suppresses dengue virus replication by down-regulation of PI3K/AKT and GRP78. Antiviral Res. 142, 158–168. doi: 10.1016/j.antiviral.2017.02.015 28238876

[B21] Chen W. C. Hossen M. Liu W. Yen C. H. Huang C. H. Hsu Y. C.. (2023). Grape seed proanthocyanidins inhibit replication of the dengue virus by targeting NF-kB and MAPK-mediated cyclooxygenase-2 expression. Viruses 15, 884. doi: 10.3390/v15040884 37112864 PMC10140912

[B22] Chen S. T. Lin Y. L. Huang M. T. Wu M. F. Cheng S. C. Lei H. Y.. (2008). CLEC5A is critical for dengue-virus-induced lethal disease. Nature 453, 672–676. doi: 10.1038/nature07013 18496526

[B23] Chen C. L. Lin C. F. Wan S. W. Wei L. S. Chen M. C. Yeh T. M.. (2013). Anti-dengue virus nonstructural protein 1 antibodies cause NO-mediated endothelial cell apoptosis via ceramide-regulated glycogen synthase kinase-3β and NF-κB activation. J. Immunol. 191, 1744–1752. doi: 10.4049/jimmunol.1201976 23851680

[B24] Chen Y. Maguire T. Hileman R. E. Fromm J. R. Esko J. D. Linhardt R. J.. (1997). Dengue virus infectivity depends on envelope protein binding to target cell heparan sulfate. Nat. Med. 3, 866–871. doi: 10.1038/nm0897-866 9256277

[B25] Chia P. Y. Thein T. L. Ong S. W. X. Lye D. C. Leo Y. S. (2020). Severe dengue and liver involvement: an overview and review of the literature. Expert Rev. Anti Infect. Ther. 18, 181–189. doi: 10.1080/14787210.2020.1720652 31971031

[B26] Chu J. J. Yang P. L. (2007). c-Src protein kinase inhibitors block assembly and maturation of dengue virus. Proc. Natl. Acad. Sci. U.S.A. 104, 3520–3525. doi: 10.1073/pnas.0611681104 17360676 PMC1805510

[B27] Chu J. J. H. Yang P. L. (2008). Pharmacological C-abl kinase inhibitors as potential anti-viral molecules for dengue virus. Int. J. Infect. Dis. 12, e297. doi: 10.1016/j.ijid.2008.05.796

[B28] Clark M. J. Miduturu C. Schmidt A. G. Zhu X. Pitts J. D. Wang J.. (2016). GNF-2 inhibits dengue virus by targeting abl kinases and the viral E protein. Cell Chem. Biol. 23, 443–452. doi: 10.1016/j.chembiol.2016.03.010 27105280 PMC4865888

[B29] Cortese M. Kumar A. Matula P. Kaderali L. Scaturro P. Erfle H.. (2019). Reciprocal effects of fibroblast growth factor receptor signaling on dengue virus replication and virion production. Cell Rep. 27, 2579–2592.e6. doi: 10.1016/j.celrep.2019.04.105 31141684

[B30] Cortese M. Mulder K. Chatel-Chaix L. Scaturro P. Cerikan B. Plaszczyca A.. (2021). Determinants in nonstructural protein 4A of dengue virus required for RNA replication and replication organelle biogenesis. J. Virol. 95, e0131021. doi: 10.1128/JVI.01310-21 34379504 PMC8513467

[B31] Cuartas-López A. M. Gallego-Gómez J. C. (2020). Glycogen synthase kinase 3ß participates in late stages of Dengue virus-2 infection. Mem Inst Oswaldo Cruz 115, e190357. doi: 10.1590/0074-02760190357 32130369 PMC7046174

[B32] Cuartas-López A. M. Hernández-Cuellar C. E. Gallego-Gómez J. C. (2018). Disentangling the role of PI3K/Akt, Rho GTPase and the actin cytoskeleton on dengue virus infection. Virus Res. 256, 153–165. doi: 10.1016/j.virusres.2018.08.013 30130602

[B33] Dankwa S. Dols M. M. Wei L. Glennon E. K. K. Kain H. S. Kaushansky A.. (2021). Exploiting polypharmacology to dissect host kinases and kinase inhibitors that modulate endothelial barrier integrity. Cell Chem. Biol. 28, 1679–1692.e4. doi: 10.1016/j.chembiol.2021.06.004 34216546 PMC8688180

[B34] Dejarnac O. Hafirassou M. L. Chazal M. Versapuech M. Gaillard J. Perera-Lecoin M.. (2018). TIM-1 ubiquitination mediates dengue virus entry. Cell Rep. 23, 1779–1793. doi: 10.1016/j.celrep.2018.04.013 29742433

[B35] de Oliveira L. C. Ribeiro A. M. Albarnaz J. D. Torres A. A. Guimarães L. F. Z. Pinto A. K.. (2020). The small molecule AZD6244 inhibits dengue virus replication in *vitro* and protects against lethal challenge in a mouse model. Arch. Virol. 165, 671–681. doi: 10.1007/s00705-020-04524-7 31942645

[B36] de Wispelaere M. LaCroix A. J. Yang P. L. (2013). The small molecules AZD0530 and dasatinib inhibit dengue virus RNA replication via Fyn kinase. J. Virol. 87, 7367–7381. doi: 10.1128/JVI.00632-13 23616652 PMC3700292

[B37] Duran A. Valero N. Mosquera J. Fuenmayor E. Alvarez-Mon M. (2017). Gefitinib and pyrrolidine dithiocarbamate decrease viral replication and cytokine production in dengue virus infected human monocyte cultures. Life Sci. 191, 180–185. doi: 10.1016/j.lfs.2017.10.027 29055802

[B38] Ferrari M. Zevini A. Palermo E. Muscolini M. Alexandridi M. Etna M. P.. (2020). Dengue virus targets nrf2 for NS2B3-mediated degradation leading to enhanced oxidative stress and viral replication. J. Virol. 94 (24), e01551–20. doi: 10.1128/JVI.01551-20 32999020 PMC7925186

[B39] Frye M. Stritt S. Ortsäter H. Hernandez Vasquez M. Kaakinen M. Vicente A.. (2020). EphrinB2-EphB4 signalling provides Rho-mediated homeostatic control of lymphatic endothelial cell junction integrity. eLife 9, e57732. doi: 10.7554/eLife.57732.sa2 32897857 PMC7478896

[B40] Gujral T. S. Peshkin L. Kirschner M. W. (2014). Exploiting polypharmacology for drug target deconvolution. Proc. Natl. Acad. Sci. U.S.A. 111, 5048–5053. doi: 10.1073/pnas.1403080111 24707051 PMC3977247

[B41] Hafirassou M. L. Meertens L. Umaña-Diaz C. Labeau A. Dejarnac O. Bonnet-Madin L.. (2017). A global interactome map of the dengue virus NS1 identifies virus restriction and dependency host factors. Cell Rep. 21, 3900–3913. doi: 10.1016/j.celrep.2017.11.094 29281836

[B42] He L. Wu S. Y. Wang T. L. Zhang P. Huang X. (2012). [Induction of VEGF in human monocytes by DENV infection and the regulatory mechanism]. Bing Du Xue Bao 28 (6), 652–657. 23367565

[B43] Hornbeck P. V. Zhang B. Murray B. Kornhauser J. M. Latham V. Skrzypek E. (2015). PhosphoSitePlus, 2014: mutations, PTMs and recalibrations. Nucleic Acids Res. 43, D512–D520. doi: 10.1093/nar/gku1267 25514926 PMC4383998

[B44] Hwang K. Yoon J. H. Lee J. H. Lee S. (2021). Recent advances in monoclonal antibody therapy for colorectal cancers. Biomedicines 9 (1), 39. doi: 10.3390/biomedicines9010039 33466394 PMC7824816

[B45] Jordan T. X. Randall G. (2017). Dengue virus activates the AMP kinase-mTOR axis to stimulate a proviral lipophagy. J. Virol. 91 (11), e02020–16. doi: 10.1128/JVI.02020-16 28298606 PMC5432877

[B46] Kao Y. S. Wang L. C. Chang P. C. Lin H. M. Lin Y. S. Yu C. Y.. (2023). Negative regulation of type I interferon signaling by integrin-linked kinase permits dengue virus replication. PLoS Pathog. 19, e1011241. doi: 10.1371/journal.ppat.1011241 36930690 PMC10057834

[B47] Kong W. Mao J. Yang Y. Yuan J. Chen J. Luo Y.. (2020). Mechanisms of mTOR and autophagy in human endothelial cell infected with dengue virus-2. Viral Immunol. 33, 61–70. doi: 10.1089/vim.2019.0009 31978319

[B48] Krishnamoorthy P. Raj A. S. Kumar P. Das N. Kumar H. (2022). Host and viral non-coding RNAs in dengue pathogenesis. Rev. Med. Virol. 32, e2360. doi: 10.1002/rmv.2360 35510480

[B49] Kumar R. Agrawal T. Khan N. A. Nakayama Y. Medigeshi G. R. (2016). Identification and characterization of the role of c-terminal Src kinase in dengue virus replication. Sci. Rep. 6, 30490. doi: 10.1038/srep30490 27457684 PMC4960526

[B50] Kumar N. Sharma S. Kumar R. Tripathi B. N. Barua S. Ly H.. (2020). Host-directed antiviral therapy. Clin. Microbiol. Rev. 33 (3), e00168–19. doi: 10.1128/CMR.00168-19 32404434 PMC7227448

[B51] Labeau A. Simon-Loriere E. Hafirassou M. L. Bonnet-Madin L. Tessier S. Zamborlini A.. (2020). A genome-wide CRISPR-cas9 screen identifies the dolichol-phosphate mannose synthase complex as a host dependency factor for dengue virus infection. J. Virol. 94 (7), e01751–19. doi: 10.1128/JVI.01751-19 31915280 PMC7081898

[B52] Lahon A. Arya R. P. Banerjea A. C. (2021). Dengue virus dysregulates master transcription factors and PI3K/AKT/mTOR signaling pathway in megakaryocytes. Front. Cell Infect. Microbiol. 11, 715208. doi: 10.3389/fcimb.2021.715208 34513730 PMC8427595

[B53] Lambeth C. R. White L. J. Johnston R. E. de Silva A. M. (2005). Flow cytometry-based assay for titrating dengue virus. J. Clin. Microbiol. 43, 3267–3272. doi: 10.1128/JCM.43.7.3267-3272.2005 16000446 PMC1169137

[B54] Lateef Z. Wise L. M. (2018). Exploitation of receptor tyrosine kinases by viral-encoded growth factors. Growth Factors 36, 118–140. doi: 10.1080/08977194.2018.1520229 31084274

[B55] Laureti M. Narayanan D. Rodriguez-Andres J. Fazakerley J. K. Kedzierski L. (2018). Flavivirus receptors: Diversity, identity, and cell entry. Front. Immunol. 9, 2180. doi: 10.3389/fimmu.2018.02180 30319635 PMC6168832

[B56] Le Sommer C. Barrows N. J. Bradrick S. S. Pearson J. L. Garcia-Blanco M. A. (2012). G protein-coupled receptor kinase 2 promotes flaviviridae entry and replication. PLoS Negl. Trop. Dis. 6, e1820. doi: 10.1371/journal.pntd.0001820 23029581 PMC3441407

[B57] Li Y. Xie J. Wu S. Xia J. Zhang P. Liu C.. (2013). Protein kinase regulated by dsRNA downregulates the interferon production in dengue virus- and dsRNA-stimulated human lung epithelial cells. PLoS One 8, e55108. doi: 10.1371/journal.pone.0055108 23372823 PMC3555826

[B58] Liang M. Li Y. Zhang K. Zhu Y. Liang J. Liu M.. (2023). Host factor DUSP5 potently inhibits dengue virus infection by modulating cytoskeleton rearrangement. Antiviral Res. 215, 105622. doi: 10.1016/j.antiviral.2023.105622 37149044

[B59] Limjindaporn T. Panaampon J. Malakar S. Noisakran S. Yenchitsomanus P. T. (2017). Tyrosine kinase/phosphatase inhibitors decrease dengue virus production in HepG2 cells. Biochem. Biophys. Res. Commun. 483, 58–63. doi: 10.1016/j.bbrc.2017.01.006 28065855

[B60] Liu Y. Chen L. Liu W. Li D. Zeng J. Tang Q.. (2021). Cepharanthine suppresses herpes simplex virus type 1 replication through the downregulation of the PI3K/akt and p38 MAPK signaling pathways. Front. Microbiol. 12. doi: 10.3389/fmicb.2021.795756 PMC8696181 34956164

[B61] Liu B. Gao T. T. Fu X. Y. Xu Z. H. Ren H. Zhao P.. (2021). PTEN lipid phosphatase activity enhances dengue virus production through akt/foxO1/maf1 signaling. Virol. Sin. 36, 412–423. doi: 10.1007/s12250-020-00291-6 33044659 PMC8257821

[B62] Liu Y. Liu H. Zou J. Zhang B. Yuan Z. (2014). Dengue virus subgenomic RNA induces apoptosis through the Bcl-2-mediated PI3k/Akt signaling pathway. Virology 448, 15–25. doi: 10.1016/j.virol.2013.09.016 24314632

[B63] Low J. G. Sung C. Wijaya L. Wei Y. Rathore A. P. S. Watanabe S.. (2014). Efficacy and safety of celgosivir in patients with dengue fever (CELADEN): a phase 1b, randomised, double-blind, placebo-controlled, proof-of-concept trial. Lancet Infect. Dis. 14, 706–715. doi: 10.1016/S1473-3099(14)70730-3 24877997

[B64] Marceau C. D. Puschnik A. S. Majzoub K. Ooi Y. S. Brewer S. M. Fuchs G.. (2016). Genetic dissection of Flaviviridae host factors through genome-scale CRISPR screens. Nature 535, 159–163. doi: 10.1038/nature18631 27383987 PMC4964798

[B65] McDermott M. S. J. Canonici A. Ivers L. Browne B. C. Madden S. F. O'Brien N. A.. (2017). Dual inhibition of IGF1R and ER enhances response to trastuzumab in HER2 positive breast cancer cells. Int. J. Oncol. 50, 2221–2228. doi: 10.3892/ijo.2017.3976 28498399

[B66] Nagila A. Netsawang J. Suttitheptumrong A. Morchang A. Khunchai S. Srisawat C.. (2013). Inhibition of p38MAPK and CD137 signaling reduce dengue virus-induced TNF-α secretion and apoptosis. Virol. J. 10, 105. doi: 10.1186/1743-422X-10-105 23557259 PMC3639879

[B67] Nasar S. Rashid N. Iftikhar S. (2020). Dengue proteins with their role in pathogenesis, and strategies for developing an effective anti-dengue treatment: A review. J. Med. Virol. 92, 941–955. doi: 10.1002/jmv.25646 31784997

[B68] Nguyen N. M. Tran C. N. Phung L. K. Duong K. T. Huynh Hle A. Farrar J.. (2013). A randomized, double-blind placebo controlled trial of balapiravir, a polymerase inhibitor, in adult dengue patients. J. Infect. Dis. 207, 1442–1450. doi: 10.1093/infdis/jis470 22807519 PMC3610419

[B69] Noppakunmongkolchai W. Poyomtip T. Jittawuttipoka T. Luplertlop N. Sakuntabhai A. Chimnaronk S.. (2016). Inhibition of protein kinase C promotes dengue virus replication. Virol. J. 13, 35. doi: 10.1186/s12985-016-0494-6 26931565 PMC4774189

[B70] Okamoto T. Suzuki T. Kusakabe S. Tokunaga M. Hirano J. Miyata Y.. (2017). Regulation of apoptosis during flavivirus infection. Viruses 9, 243. doi: 10.3390/v9090243 28846635 PMC5618009

[B71] Osuna-Ramos J. F. Reyes-Ruiz J. M. del Ángel R. M. (2018). The role of host cholesterol during flavivirus infection. Front. Cell. Infection Microbiol. 8. doi: 10.3389/fcimb.2018.00388 PMC6224431 30450339

[B72] Palanichamy Kala M. St John A. L. Rathore A. P. S. (2023). Dengue: Update on clinically relevant therapeutic strategies and vaccines. Curr. Treat Options Infect. Dis. 15, 27–52. doi: 10.1007/s40506-023-00263-w 37124673 PMC10111087

[B73] Pelletier J. P. R. Mukhtar F. (2020). Passive monoclonal and polyclonal antibody therapies. Immunologic Concepts in Transfusion Medicine 2020, 251–348. doi: 10.1016/B978-0-323-67509-3.00016-0

[B74] Pozzi B. Bragado L. Mammi P. Torti M. F. Gaioli N. Gebhard L. G.. (2020). Dengue virus targets RBM10 deregulating host cell splicing and innate immune response. Nucleic Acids Res. 48, 6824–6838. doi: 10.1093/nar/gkaa340 32432721 PMC7337517

[B75] Pu S. Y. Xiao F. Schor S. Bekerman E. Zanini F. Barouch-Bentov R.. (2018). Feasibility and biological rationale of repurposing sunitinib and erlotinib for dengue treatment. Antiviral Res. 155, 67–75. doi: 10.1016/j.antiviral.2018.05.001 29753658 PMC6064211

[B76] Puerta-Guardo H. Biering S. B. de Sousa F. T. G. Shu J. Glasner D. R. Li J.. (2022). Flavivirus NS1 triggers tissue-specific disassembly of intercellular junctions leading to barrier dysfunction and vascular leak in a GSK-3β-dependent manner. Pathogens 11 (6), 615. doi: 10.3390/pathogens11060615 35745469 PMC9228372

[B77] Rahman M. A. Shorobi F. M. Uddin M. N. Saha S. Hossain M. A. (2022). Quercetin attenuates viral infections by interacting with target proteins and linked genes in chemicobiological models. In Silico Pharmacol. 10, 17. doi: 10.1007/s40203-022-00132-2 36119653 PMC9477994

[B78] Roskoski R. Jr. (2023). Properties of FDA-approved small molecule protein kinase inhibitors: A 2023 update. Pharmacol. Res. 187, 106552. doi: 10.1016/j.phrs.2022.106552 36403719

[B79] Roy S. K. Bhattacharjee S. (2021). Dengue virus: epidemiology, biology, and disease aetiology. Can. J. Microbiol. 67, 687–702. doi: 10.1139/cjm-2020-0572 34171205

[B80] Salokas K. Liu X. Öhman T. Chowdhury I. Gawriyski L. Keskitalo S.. (2022). Physical and functional interactome atlas of human receptor tyrosine kinases. EMBO Rep. 23 (6), e54041. doi: 10.15252/embr.202154041 35384245 PMC9171411

[B81] Savidis G. McDougall W. M. Meraner P. Perreira J. M. Portmann J. M. Trincucci G.. (2016). Identification of zika virus and dengue virus dependency factors using functional genomics. Cell Rep. 16, 232–246. doi: 10.1016/j.celrep.2016.06.028 27342126

[B82] Shannon P. Markiel A. Ozier O. Baliga N. S. Wang J. T. Ramage D.. (2003). Cytoscape: a software environment for integrated models of biomolecular interaction networks. Genome Res. 13, 2498–2504. doi: 10.1101/gr.1239303 14597658 PMC403769

[B83] Shyu H. W. Lin Y. Y. Chen L. C. Wang Y. F. Yeh T. M. Su S. J.. (2010). The dengue virus envelope protein induced PAI-1 gene expression via MEK/ERK pathways. Thromb. Haemost. 104, 1219–1227. doi: 10.1160/TH10-05-0302 20886187

[B84] Silva J. M. McMahon M. (2014). The fastest Western in town: a contemporary twist on the classic Western blot analysis. J. Vis. Exp. 84, e51149. doi: 10.3791/51149 PMC4028330 24561642

[B85] Singh S. Anupriya M. G. Modak A. Sreekumar E. (2018). Dengue virus or NS1 protein induces trans-endothelial cell permeability associated with VE-Cadherin and RhoA phosphorylation in HMEC-1 cells preventable by Angiopoietin-1. J. Gen. Virol. 99, 1658–1670. doi: 10.1099/jgv.0.001163 30355397

[B86] Sinha M. Chakraborty U. Kool A. Chakravarti M. Das S. Ghosh S.. (2022). *In-vitro* antiviral action of Eupatorium perfoliatum against dengue virus infection: Modulation of mTOR signaling and autophagy. J. Ethnopharmacol 282, 114627. doi: 10.1016/j.jep.2021.114627 34509603

[B87] Smith J. L. Jeng S. McWeeney S. K. Hirsch A. J. (2017). A microRNA screen identifies the wnt signaling pathway as a regulator of the interferon response during flavivirus infection. J. Virol. 91 (8), e02388–16. doi: 10.1128/JVI.02388-16 28148804 PMC5375670

[B88] Sreekanth G. P. Chuncharunee A. Cheunsuchon B. Noisakran S. Yenchitsomanus P. T. Limjindaporn T. (2017). JNK1/2 inhibitor reduces dengue virus-induced liver injury. Antiviral Res. 141, 7–18. doi: 10.1016/j.antiviral.2017.02.003 28188818

[B89] Sreekanth G. P. Chuncharunee A. Sirimontaporn A. Panaampon J. Noisakran S. Yenchitsomanus P. T.. (2016). SB203580 modulates p38 MAPK signaling and dengue virus-induced liver injury by reducing MAPKAPK2, HSP27, and ATF2 phosphorylation. PLoS One 11, e0149486. doi: 10.1371/journal.pone.0149486 26901653 PMC4764010

[B90] Sreekanth G. P. Chuncharunee A. Sirimontaporn A. Panaampon J. Srisawat C. Morchang A.. (2014). Role of ERK1/2 signaling in dengue virus-induced liver injury. Virus Res. 188, 15–26. doi: 10.1016/j.virusres.2014.03.025 24704674

[B91] Sreekanth G. P. Chuncharunee A. Yenchitsomanus P. T. Limjindaporn T. (2020). Crocetin improves dengue virus-induced liver injury. Viruses 12 (8), 825. doi: 10.3390/v12080825 32751420 PMC7472398

[B92] Sreekanth G. P. Yenchitsomanus P. T. Limjindaporn T. (2018). Role of mitogen-activated protein kinase signaling in the pathogenesis of dengue virus infection. Cell Signal 48, 64–68. doi: 10.1016/j.cellsig.2018.05.002 29753850

[B93] Suksanpaisan L. Susantad T. Smith D. R. (2009). Characterization of dengue virus entry into HepG2 cells. J. BioMed. Sci. 16, 17. doi: 10.1186/1423-0127-16-17 19272179 PMC2653518

[B94] Tian Y. S. Zhou Y. Takagi T. Kameoka M. Kawashita N. (2018). Dengue virus and its inhibitors: A brief review. Chem. Pharm. Bull. (Tokyo) 66, 191–206. doi: 10.1248/cpb.c17-00794 29491253

[B95] Trenker R. Jura N. (2020). Receptor tyrosine kinase activation: From the ligand perspective. Curr. Opin. Cell Biol. 63, 174–185. doi: 10.1016/j.ceb.2020.01.016 32114309 PMC7813211

[B96] Tricou V. Minh N. N. Van T. P. Lee S. J. Farrar J. Wills B.. (2010). A randomized controlled trial of chloroquine for the treatment of dengue in Vietnamese adults. PLoS Negl. Trop. Dis. 4, e785. doi: 10.1371/journal.pntd.0000785 20706626 PMC2919376

[B97] Troost B. Smit J. M. (2020). Recent advances in antiviral drug development towards dengue virus. Curr. Opin. Virol. 43, 9–21. doi: 10.1016/j.coviro.2020.07.009 32795907

[B98] Udawatte D. J. Lang D. M. Currier J. R. Medin C. L. Rothman A. L. (2022). Dengue virus downregulates TNFR1- and TLR3-stimulated NF-κB activation by targeting RIPK1. Front. Cell. Infection Microbiol. 12. doi: 10.3389/fcimb.2022.926036 PMC9615918 36310878

[B99] Valencia H. J. de Aguiar M. Costa M. A. Mendonça D. C. Reis E. V. Arias N. E. C.. (2021). Evaluation of kinase inhibitors as potential therapeutics for flavivirus infections. Arch. Virol. 166, 1433–1438. doi: 10.1007/s00705-021-05021-1 33683474 PMC7938686

[B100] Vincetti P. Caporuscio F. Kaptein S. Gioiello A. Mancino V. Suzuki Y.. (2015). Discovery of multitarget antivirals acting on both the dengue virus NS5-NS3 interaction and the host src/fyn kinases. J. Med. Chem. 58, 4964–4975. doi: 10.1021/acs.jmedchem.5b00108 26039671

[B101] Wang K. Wang J. Sun T. Bian G. Pan W. Feng T.. (2016). Glycosphingolipid GM3 is indispensable for dengue virus genome replication. Int. J. Biol. Sci. 12, 872–883. doi: 10.7150/ijbs.15641 27313500 PMC4910605

[B102] Wei L. Dankwa S. Vijayan K. Smith J. D. Kaushansky A. (2022). Temporally resolved kinase regulatory networks control endothelial barrier integrity. bioRxiv. doi: 10.1101/2022.09.19.508598 PMC10927359 38467420

[B103] Whitehorn J. Nguyen C. V. V. Khanh L. P. Kien D. T. H. Quyen N. T. H. Tran N. T. T.. (2016). Lovastatin for the treatment of adult patients with dengue: A randomized, double-blind, placebo-controlled trial. Clin. Infect. Dis. 62, 468–476. doi: 10.1093/cid/civ949 26565005 PMC4725386

[B104] Wu N. Gou X. Hu P. Chen Y. Ji J. Wang Y.. (2022). Mechanism of autophagy induced by activation of the AMPK/ERK/mTOR signaling pathway after TRIM22-mediated DENV-2 infection of HUVECs. Virol. J. 19, 228. doi: 10.1186/s12985-022-01932-w 36587218 PMC9805691

[B105] Xia Z. Ren Y. Li S. Xu J. Wu Y. Cao Z. (2021). ML-SA1 and SN-2 inhibit endocytosed viruses through regulating TRPML channel expression and activity. Antiviral Res. 195, 105193. doi: 10.1016/j.antiviral.2021.105193 34687820

[B106] Yang X. Quam M. B. M. Zhang T. Sang S. (2021). Global burden for dengue and the evolving pattern in the past 30 years. J. Travel Med. 28 (8), taab146. doi: 10.1093/jtm/taab146 34510205

[B107] Ye H. Duan X. Yao M. Kang L. Li Y. Li S.. (2021). USP18 mediates interferon resistance of dengue virus infection. Front. Microbiol. 12. doi: 10.3389/fmicb.2021.682380 PMC8130619 34017322

[B108] Yeh T. M. Liu S. H. Lin K. C. Kuo C. Kuo S. Y. Huang T. Y.. (2013). Dengue virus enhances thrombomodulin and ICAM-1 expression through the macrophage migration inhibitory factor induction of the MAPK and PI3K signaling pathways. PLoS One 8, e55018. doi: 10.1371/journal.pone.0055018 23383040 PMC3557271

[B109] Yuan K. Chen Y. Zhong M. Lin Y. Liu L. (2022). Risk and predictive factors for severe dengue infection: A systematic review and meta-analysis. PLoS One 17, e0267186. doi: 10.1371/journal.pone.0267186 35427400 PMC9012395

[B110] Zeng Z. Zhan J. Chen L. Chen H. Cheng S. (2021). Global, regional, and national dengue burden from 1990 to 2017: A systematic analysis based on the global burden of disease study 2017. EClinicalMedicine 32, 100712. doi: 10.1016/j.eclinm.2020.100712 33681736 PMC7910667

[B111] Zhang R. Miner J. J. Gorman M. J. Rausch K. Ramage H. White J. P.. (2016). A CRISPR screen defines a signal peptide processing pathway required by flaviviruses. Nature 535, 164–168. doi: 10.1038/nature18625 27383988 PMC4945490

