# Supplementary figures and images for: Multiple receptor tyrosine kinases regulate dengue infection of hepatocytes
Source: Front Cell Infect Microbiol. 2024 Mar 22;14:1264525. doi: 10.3389/fcimb.2024.1264525 (PMC10995305; doi:10.3389/fcimb.2024.1264525)

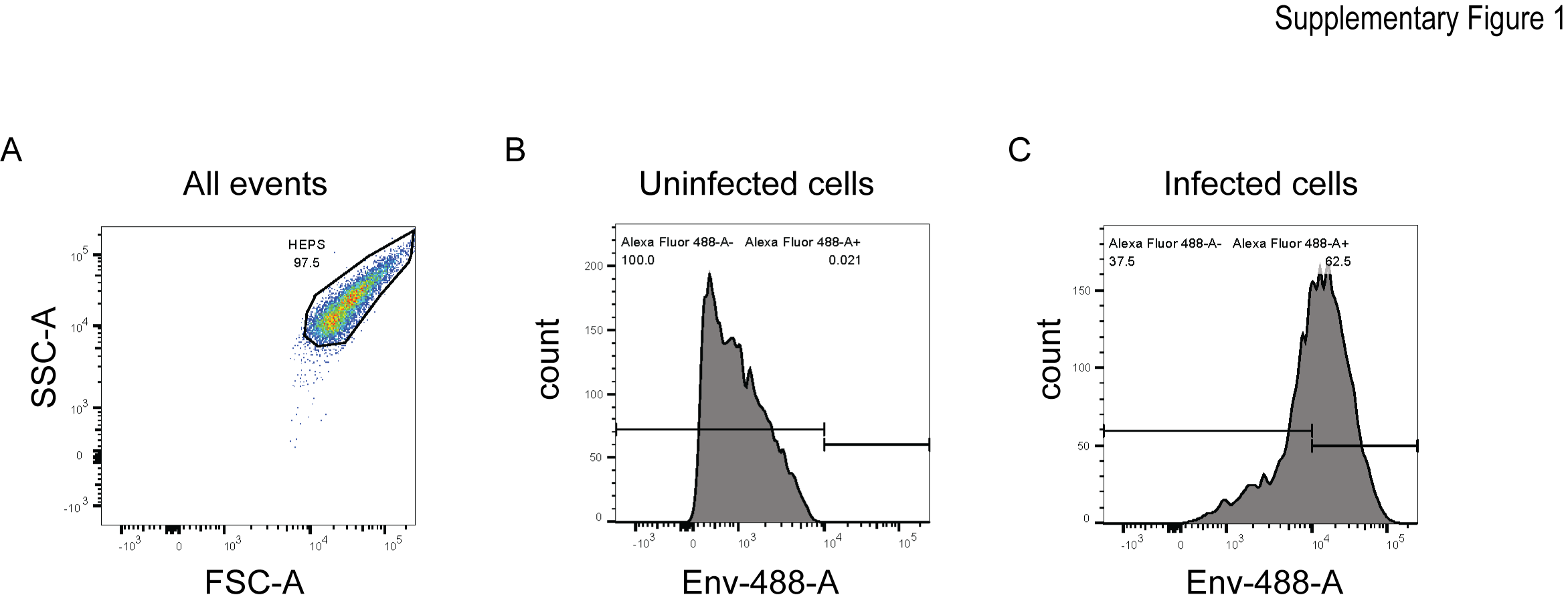

Supplement: Supplementary file 1 [file Image_1.tif]

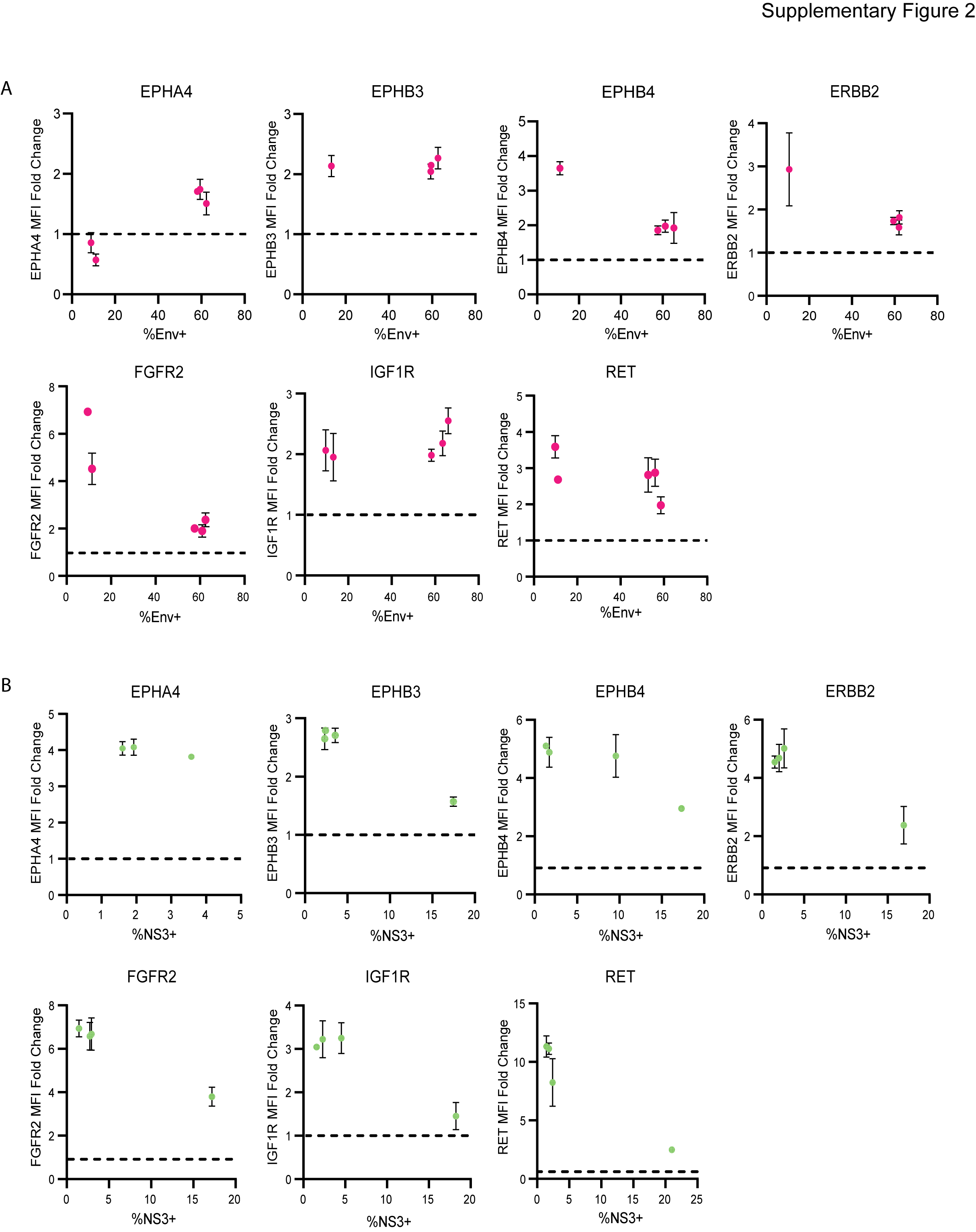

Supplement: Supplementary file 2 [file Image_2.tif]

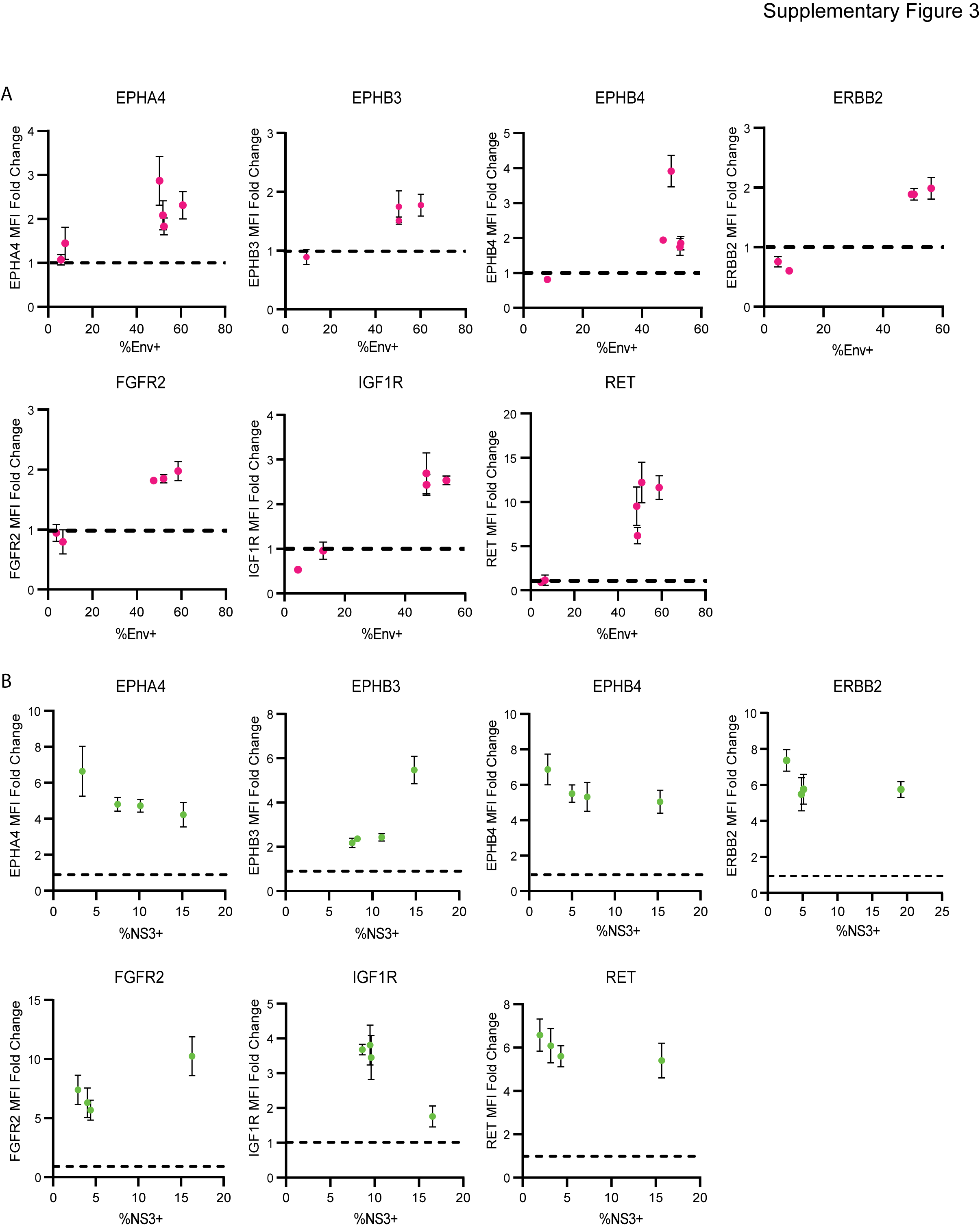

Supplement: Supplementary file 3 [file Image_3.tif]

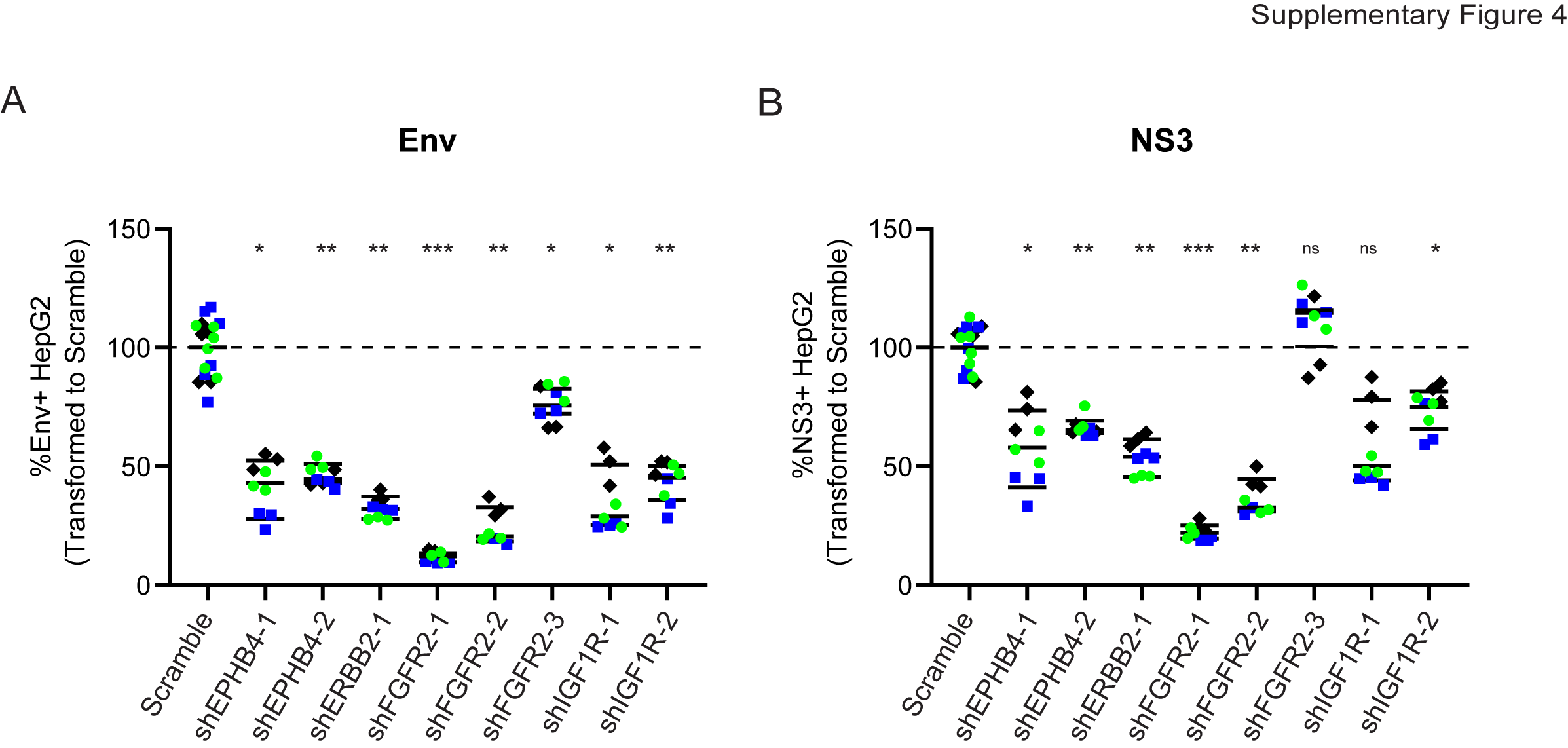

Supplement: Supplementary file 4 [file Image_4.tif]

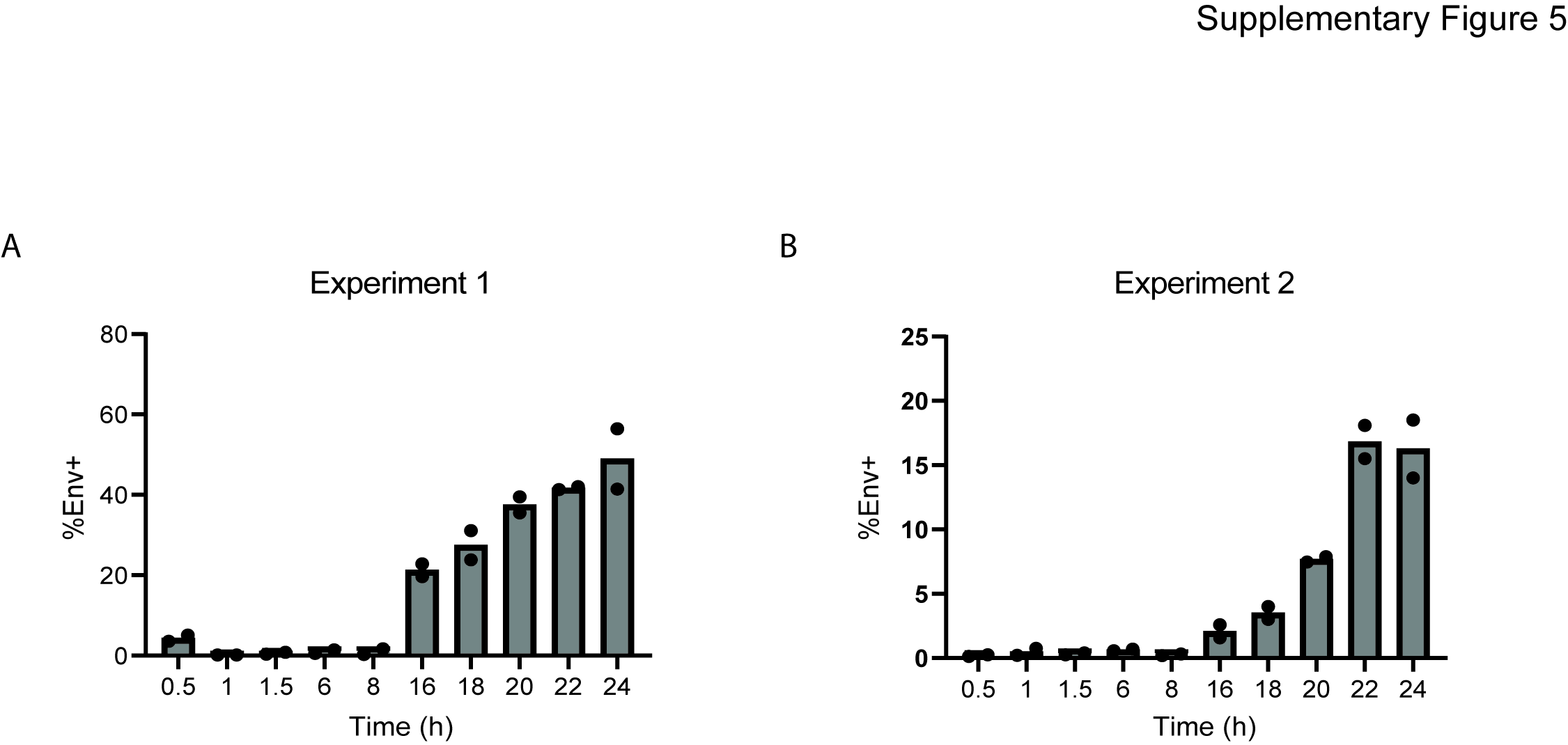

Supplement: Supplementary file 5 [file Image_5.tif]
