# Supplementary material for: Multiple receptor tyrosine kinases regulate dengue infection of hepatocytes
Source: Front Cell Infect Microbiol. 2024 Mar 22;14:1264525. doi: 10.3389/fcimb.2024.1264525 (PMC10995305; doi:10.3389/fcimb.2024.1264525)
Supplement: Supplementary file 7 [file DataSheet_2.pdf]

Supplementary Information

Supplementary Figure 1 Flow gating strategy for DENV infection in HepG2 cells

DENV infection is quantified by gating for cells by size (FSC-A x SSC-A) then measuring the percent of cells staining positive for DENV envelope (Env) protein or non-structural protein 3 (NS3).

Supplementary Figure 2 Difference in total RTK expression in DENV-infected cells across biological replicates with varying infection rates

The fold change in total RTK expression between bystander and infected cells is shown against the average infection rate for each experiment for Env (A) and NS3 (B). Dashed line denotes fold change = 1 to draw attention to the difference in scale for fold change across the RTKs.

Supplementary Figure 3 Difference in surface RTK expression in DENV-infected cells across biological replicates with varying infection rates

The fold change in surface RTK expression between bystander and infected cells is shown against the average infection rate for each experiment for Env (A) and NS3 (B). Dashed line denotes fold change = 1 to draw attention to the difference in scale for fold change across the RTKs.

Supplementary Figure 4 Effect of RTK knockdown on Env+ or NS3+ cells

Knockdown of EPHB4, ERBB2, FGFR2, and IGF1R results in decreased DENV infection. HepG2 cells were transduced with shRNA lentivirus targeting KiR-predicted RTKs then analyzed for protein reduction by western blot (A-B). Viable cells with successful knockdown in protein expression were infected with DENV2 MON601, and the percent change of Env+ (A) and NS3+ (B) cells is shown. Data represent three independent infections on a single transduction per clone. Significance of differences in infection between knockdown lines and scramble were analyzed by Student's t-test and are indicated on the graph, where ns = non-significant, * = p-value<0.05, ** = p-value<0.05, and *** = p-value<0.0005.

Supplementary Figure 5 Temporal phosphorylation response to DENV infection

HepG2 cells were infected with DENV2 then harvested at the indicated time-point to quantify infection by flow cytometry (in parallel with p-ERBB2 and -IGF1R analysis [Figure 5]). Cells were fixed, permeabilized, blocked then stained with anti-Env-488. Percent of Env+ cells for two independent experiments are shown (A-B).

Supplementary Tables

Supplementary Table 1 lists the kinase inhibitors used in the KiR screen with corresponding CAS number. All inhibitors were used at 500 nM.

**Supplementary Table 1 Kinase Regression Inhibitor Panel**	
Inhibitor ID	*CAS #*

Aminopurvanolol A	220792-57-4
AMPK Inhibitor; Compound C (Dorsomorphin)	866405-64-3
Bosutinib	380843-75-4
Casein kinase I inhibitor D4476	301836-43-1
Cdk1/2 Inhibitor III	443798-55-8
CDK2 inhibitor IV; NU6140	444723-13-1
CDK4 inhibitor	546102-60-7
Dasatinib	302962-49-8
Dovitinib	405169-16-6
EGFR/ErbB2/ErbB4 inhibitor	881001-19-0
Erlotinib	183321-74-6
Gefitinib	184475-35-2
Go 6976	136194-77-9
Go 6983	133053-19-7
GSK inhibitor IX (BIO)	667463-62-9
GSK-3 Inhibitor X	740841-15-0
GSK-3 Inhibitor XIII	404828-08-6
GSK-3b inhibitor I (TDZD-6)	327036-89-5
H89	130964-39-5
Imatinib	152459-95-5
JAK inhibitor I	457081-03-7
JNK inhibitor II (SP600125)	129-56-6

K252a	99533-80-9
Lapatinib	388082-78-8
Lck inhibitor	213743-31-8
Masitinib	790299-79-5
Nilotinib	641571-10-0
PKR inhibitor	608512-97-6
ROCK inhibitor (Y-27632)	129830-38-2
SB218078	135897-06-2
Sorafenib	284461-73-0
Staurosporine	62996-74-1
Staurosporine n benzoyl	120685-11-2
SU11274	658084-23-2
SU6656	330161-87-0
Tofacitinib	477600-75-2
TWS119	601514-19-6
Vandetanib	443913-73-3

Supplementary Table 2 lists the kinases predicted by KiR on dengue infection in hepatocytes with the corresponding coefficient of correlation at alpha = 0.8. Positive coefficient of correlation indicates the kinase is predicted to promote DENV infection, negative coefficient of correlation indicates the kinase is predicted to restrict DENV infection.

Supplementary Table 2 KiR-predicted Kinases Regulating DENV Infection	
Predicted Kinase	*Coefficient of Correlation*
ACK1	-0.04048
CHK1	-0.02247
CK1g3	-0.05048
CTK_MATK	-0.00949
DYRK4	0.294582
EPHA4	0.020529
EPHB3	0.020855
EPHB4	0.009052
ERBB2/HER2	0.076245
ERK1	-0.129
FGFR2	-0.0591
HIPK1	-0.06499
IGF1R	0.294032
IKKa/CHUK	0.251537
JAK3	0.07556
KHS_MAP4K5	0.048963
LKB1	-0.01933
MAPKAPK5/PRAK	0.171559
MARK1	0.00079
MARK4	0.022757
NEK11	0.036702

NEK3	-0.03688
NIK/MAP3K14	0.013453
P38b/MAPK11	0.014872
P38d/MAPK13	-0.02163
PAK1	0.178078
PAK4	-0.0126
PAK5	-0.00049
PIM3	0.066172
PKCepsilon	-0.01607
PKG1a	0.039417
RET	-0.03284
ROCK1	-0.00073
SIK2	0.10975
SRPK1	0.016612
TTK	-0.01278

Supplementary Table 3 details the shRNA constructs used to generate kinase knockdown cells. Scrambled control was obtained from Sigma-Aldrich® (# SCH002).

**Supplementary Table 3 MISSION shRNA Constructs**							
Clone ID	Oligo Seq	RefSeq ID	Gene ID	Taxon ID	Gene Description	Validated?	Validated Cell Line

TRCN 00000 10165	CCGGTCA GTCCGTG TGTTCTA TAAACTC GAGTTTA TAGAACA CACGGAC TGATTTT T	NM_00 4438.3	2043	9606	EPH receptor A4	Yes	A549
TRCN 00000 06427	CCGGCC CAAACCT CTTCATA TTGAACT CGAGTTC AATATGA AGAGGTT TGGGTTT TT	NM_00 4443.3	2049	9606	EPH receptor B3	Yes	MCF7
TRCN 00000 06428	CCGGGC AGTACAT TGCTCCT GGAATCT CGAGATT	NM_00 4443.3	2049	9606	EPH receptor B3	Yes	MCF7

	CCAGGA GCAATGT ACTGCTT TTT						
TRCN 00000 01773	CCGGCAA TGGGAGA GAAGCAG AATACTC GAGTATT CTGCTTC TCTCCCA TTGTTTTT	NM_00 4444.4	2050	9606	EPH receptor B4	Yes	A549
TRCN 00000 01774	CCGGTGA TCTGAAG TGGGTGA CATTCTC GAGAATG TCACCCA CTTCAGA TCATTTTT	NM_00 4444.4	2050	9606	EPH receptor B4	Yes	A549
TRCN 00000 39878	CCGGTGT CAGTATC CAGGCTT	NM_00 100586 2.1,NM	2064	9606	v-erb-b2 erythroblas tic	Yes	A549

	TGTACTC GAGTACA AAGCCTG GATACTG ACATTTT TG	_00444 8.2			leukemia viral oncogene homolog 2, neuro/gliob lastoma derived oncogene homolog (avian)		
TRCN 00000 39881	CCGGCA GTGCCAA TATCCAG GAGTTCT CGAGAAC TCCTGGA TATTGGC ACTGTTT TTG	NM_00 100586 2.1,NM _00444 8.2	2064	9606	v-erb-b2 erythroblas tic leukemia viral oncogene homolog 2, neuro/gliob lastoma derived oncogene homolog (avian)	Yes	A549

TRCN0000000366	CCGGGCACACACTTACAGAGCACAACTCGAGTTGTGCTCTGTAAGTGTGTGCTTTTT	NM_000141.4, NM_001144914.1,NM_001144915.1, NM_001144916.1,NM_001144917.1, NM_001144918.1,NM_022970.3	2263	9606	fibroblast growth factor receptor 2		
TRCN0000000367	CCGGGCCACCAACCAAATACCAAATCTCGAGATTTGGTATT	NM_000141.4, NM_001144913.1,NM_00114	2263	9606	fibroblast growth factor receptor 2		

	TGGTTGG TGGCTTT TT	4914.1, NM_00 114491 7.1,NM _02297 0.3					
TRCN 00000 00368	CCGGCC GAATGAA GAACACG ACCAACT CGAGTTG GTCGTGT TCTTCAT TCGGTTT TT	NM_00 0141.4, NM_00 114491 3.1,NM _00114 4914.1, NM_00 114491 5.1,NM _00114 4916.1, NM_00 114491 8.1,NM _00114 4919.1,	2263	9606	fibroblast growth factor receptor 2		

		NM_022970.3					
TRCN0000000424	CCGGGCTGATGTGTACGTTCCTGATCTCGAGATCAGGAACGTACACATCAGCTTTTT	NM_000875.3	3480	9606	insulin-like growth factor 1 receptor	Yes	A549
TRCN0000000425	CCGGCCTTGGACGTTCTTTCAGCATCTCGAGATGCTGAAAGAACGTCCAAGGTTTTT	NM_000875.3	3480	9606	insulin-like growth factor 1 receptor	Yes	A549
TRCN0000000404	CCGGCCGCTGGTGGACTGTA	NM_020630.4,	5979	9606	ret proto-oncogene	Yes	MCF7

	ATAATCTCGAGATTATTACAGTCCACCAGCGGTTTTT	NM_020975.4					
TRCN0000000405	CCGGGCTGCATGAGAACAACTGGATCTCGAGATCCAGTTGTTCTCATGCAGCTTTTT	NM_020630.4, NM_020975.4	5979	9606	ret proto-oncogene	Yes	MCF7

Supplementary Files

Supplementary File 1 “KinaseRegression.ipynb” includes the code used to run KiR on DENV infection.

Supplementary File 2 “Figure1B-C_KinasePrediction_alpharange.txt” includes the KiR output for DENV infection across the range of alphas.

Supplementary File 3 “Figure1D.txt” includes the L2 phosphosignaling network from KiR input into Cytoscape to generate Figure 1D.

Supplementary File 4 “Figure1B_raw.xlsx” includes the raw data used to graph Figure 1B.

Supplementary File 5 “Figure 5_raw.xlsx” includes the raw data used to graph Figure 5C-D.
